# Clinical significance of plasma-free amino acids and tryptophan metabolites in patients with non-small cell lung cancer receiving PD-1 inhibitor: a pilot cohort study for developing a prognostic multivariate model

**DOI:** 10.1136/jitc-2021-004420

**Published:** 2022-05-11

**Authors:** Koichi Azuma, Huihui Xiang, Tomoyuki Tagami, Rika Kasajima, Yumiko Kato, Sachise Karakawa, Shinya Kikuchi, Akira Imaizumi, Norikazu Matsuo, Hidenobu Ishii, Takaaki Tokito, Akihiko Kawahara, Kenta Murotani, Tetsuro Sasada, Yohei Miyagi, Tomoaki Hoshino

**Affiliations:** 1 Division of Respirology, Neurology, and Rheumatology, Department of Internal Medicine, Kurume University School of Medicine, Kurume, Japan; 2 Molecular Pathology and Genetics Division, Kanagawa Cancer Center Research Institute, Yokohama, Japan; 3 Research Institute for Bioscience Products and Fine Chemicals, Ajinomoto Co Inc, Kawasaki, Japan; 4 Department of Diagnostic Pathology, Kurume University Hospital, Kurume, Japan; 5 Biostatistics Center, Kurume University School of Medicine, Kurume, Japan; 6 Division of Cancer Immunotherapy, Kanagawa Cancer Center Research Institute, Yokohama, Japan; 7 Division of Respirology, Neurology, and Rheumatology Department of Internal Medicine, Kurume University School of Medicine, Kurume, Japan

**Keywords:** Lung Neoplasms, Programmed Cell Death 1 Receptor, Translational Medical Research, Tumor Biomarkers, Immunotherapy

## Abstract

**Background:**

Amino acid metabolism is essential for tumor cell proliferation and regulation of immune cell function. However, the clinical significance of free amino acids (plasma-free amino acids (PFAAs)) and tryptophan-related metabolites in plasma has not been fully understood in patients with non-small cell lung cancer (NSCLC) who receive immune checkpoint inhibitors.

**Methods:**

We conducted a single cohort observational study. Peripheral blood samples were collected from 53 patients with NSCLC before treatment with PD-1 (Programmed cell death-1) inhibitors. The plasma concentrations of 21 PFAAs, 14 metabolites, and neopterin were measured by liquid chromatography–mass spectrometry. Using Cox hazard analysis with these variables, a multivariate model was established to stratify patient overall survival (OS). Gene expression in peripheral blood mononuclear cells (PBMCs) was compared between the high-risk and low-risk patients by this multivariate model.

**Results:**

On Cox proportional hazard analysis, higher concentrations of seven PFAAs (glycine, histidine, threonine, alanine, citrulline, arginine, and tryptophan) as well as lower concentrations of three metabolites (3h-kynurenine, anthranilic acid, and quinolinic acid) and neopterin in plasma were significantly correlated with better OS (p<0.05). In particular, the multivariate model, composed of a combination of serine, glycine, arginine, and quinolinic acid, could most efficiently stratify patient OS (concordance index=0.775, HR=3.23, 95% CI 2.04 to 5.26). From the transcriptome analysis in PBMCs, this multivariate model was significantly correlated with the gene signatures related to immune responses, such as CD8 T-cell activation/proliferation and proinflammatory immune responses, and 12 amino acid-related genes were differentially expressed between the high-risk and low-risk groups.

**Conclusions:**

The multivariate model with PFAAs and metabolites in plasma might be useful for stratifying patients who will benefit from PD-1 inhibitors.

**Key messages**

**What is already known on this topic**

- Imbalances of plasma-free amino acids (PFAAs) and related metabolites have been reported in various types of cancer, but their impact on antitumor immune responses has not been fully understood.

**What this study adds**

- The multivariate model, composed of a combination of serine, glycine, arginine, and quinolinic acid, could efficiently stratify overall survival in patients with non-small cell lung cancer who receive PD-1 inhibitors. This model was significantly correlated with the immune-related gene signatures in peripheral blood mononuclear cells.

**How this study might affect research, practice or policy**

- The profiling of PFAAs and metabolites in plasma might be useful for stratifying patients with cancer who will benefit from PD-1 inhibitors.

## Introduction

Immune checkpoint inhibitors (ICIs) have provided new therapeutic approaches for treatment of otherwise incurable cancer patients. Recently, ICI therapy has become widely accepted as a standard cancer treatment for various cancers, including non-small cell lung cancer (NSCLC). However, only a limited number of patients show long-term clinical benefits. Thus, biomarkers for patient selection need to be developed to identify those who will optimally benefit from ICI and to avoid unnecessary adverse events (AEs) and treatment costs.

To date, several biomarkers, such as PD-L1 (Programmed death-Ligand 1) expression in tumor or immune cells, the existence of driver gene mutations, and tumor mutational burden, have been available to predict patients’ responses to ICI but with limited accuracy.^1^ Further, various next-generation ‘omics’ technologies that evaluate the genome, transcriptome, and epigenome have been applied to development of novel biomarkers,^2^ but reliable discrimination technology is yet to be determined. In addition to good discrimination performance, simple and less invasive techniques that do not require tumor tissues remain to be developed because tumor samples often cannot be easily obtained especially in patients with cancer without indication of surgical treatment. Therefore, blood testing might be one of the desirable approaches for biomarker development.^3^

The regulation of amino acid metabolism has been reported to be essential for tumor cell proliferation and regulation of immune cell functions.^4^ Thus, the amino acid profiles could be a critical marker to evaluate metabolic reprogramming in the tumor immune microenvironment and the metabolic condition of systemic immune cells. The amino acid profiles that show characteristic changes in different cancers can be used for diagnosis and evaluation of prognosis after tumor resection.^5 6^ The present study thus aimed to evaluate the clinical significance of plasma-free amino acid (PFAA) and tryptophan metabolites to ultimately develop a reliable method for stratifying patients with cancer who might benefit from ICI therapy.

## Materials and methods

### Patients

This study was a single cohort observational study that included patients with advanced or recurrent NSCLC who were scheduled to receive anti-PD-1 antibody (nivolumab or pembrolizumab) without concurrent chemotherapy at Kurume University Hospital (Fukuoka, Japan) between May 2017 and September 2019. As an exploratory study, we set an eligible target number of cases during the study period. All eligible patients received nivolumab (3 mg/kg/body) every 2 weeks or pembrolizumab (200 mg/body) every 3 weeks. Kidney dysfunction, liver cirrhosis, and congenital metabolic disorder were excluded.

### Data collection

All clinical data, including blood test results, were collected from the patients’ medical records. The best overall response was defined as the best response designation recorded between the date of the first dose of PD-1 inhibitor and the date of the first documented tumor progression. Treatment response was determined according to the Response Evaluation Criteria in Solid Tumors (RECIST) v.1.1. Progression-free survival (PFS) was defined as the period from the date of the first dose to the date of disease progression or death due to any cause. Overall survival (OS) was defined as the period from the date of the first dose to the date of death from any cause. The severity of AEs was graded using the National Cancer Institute Common Terminology Criteria for Adverse Events V.4.0. PD-L1 expression was examined by immunohistochemistry with anti-PD-L1 antibody (clone E1L3N; Cell Signaling Technology, Danvers, Massachusetts, USA) in paraffin-embedded tissue samples, as reported previously.^7^

### Analysis of PFAAs

Peripheral blood samples were collected in the morning before initiation of ICI therapy. After an overnight fast, blood samples (5 mL) were collected from the antecubital vein into tubes containing EDTA-2Na as an anticoagulant and were immediately (<1 min) placed on ice. Plasma was separated from the whole blood samples via centrifugation at 3000 rpm (at 1000G or more to reduce platelet contamination) for 15 min at 4°C and stored at −80°C until analysis. After thawing, plasma samples were deproteinized using acetonitrile at a final concentration of 50% before measuring amino acid concentrations using high-performance liquid chromatography–electrospray ionization mass spectrometry via precolumn derivatization, as described previously.^8^ This deproteinizing step with acetonitrile allowed analysis of amino acids bound to protein carriers in plasma. Concentrations of the following 21 amino acids were measured: alanine, alpha-amino-butyric acid, arginine, asparagine, citrulline, glutamine, glutamate, glycine, histidine, isoleucine, leucine, lysine, methionine, ornithine, phenylalanine, proline, serine, threonine, tryptophan, tyrosine, and valine.

### Analysis of tryptophan-related metabolites and neopterin in plasma

Tryptophan-related metabolites and neopterin were analyzed as reported previously.^9^ Briefly, 14 metabolites (3-hydroxyanthranilic acid, 3-hydroxy-kynurenine, 3-indoleacetic acid, 5-hydroxyindole-3-acetic acid, 5-hydroxy-L-tryptophan, anthranilic acid, indole-3-lactic acid, kynurenine, kynurenic acid, picolinic acid, quinolinic acid, serotonin, xanthurenic acid, and N′-formyl-kynurenine) in deproteinized plasma were analyzed using liquid chromatography–electrospray ionization tandem mass spectrometry. These metabolites were successfully separated within 15 min without derivatization using a reversed-phase pentafluorophenyl column for liquid chromatography separation. Neopterin, which is known as a biomarker for inflammation and is often evaluated with tryptophan metabolites, was also simultaneously analyzed.

### Statistical analysis for developing multivariate models

To develop the multivariate model according to previously reported methods,^10^ the PFAAs and metabolites associated with OS (p<0.1) without adjusting for multiplicity were chosen as variables for multivariate regression analysis with the Cox proportional hazards model. Stepwise variable selection was performed to minimize the Akaike information criterion (AIC) and the concordance index (C-index), and its 95% CI was also estimated. To verify the combination of variables, leave-one-out cross-validation (LOOCV) was applied to the multivariate model using the same variables set. In brief, one matched set composed of one responder patient, and a corresponding non-responder patient was omitted from the training data set. The logistic regression model was then calculated using the remaining samples to re-estimate coefficients for each variable. The function values for the left-out matched set were calculated based on this model. This process was repeated until every sample in the study data set had been left out once. Cut-off values of the multivariate model for Kaplan-Meier analysis and log-rank test were established for the first quartile, median, and third quartile values. The HR and 95% CI between the stratified groups with a multivariate model were determined using Cox proportional hazard analysis. Complete-case analysis was performed in the development of the multivariate model.

### Preparation of peripheral blood mononuclear cells (PBMCs) and total RNA extraction

PBMCs were isolated by density gradient centrifugation using Ficoll-Paque Plus (GE Healthcare, Uppsala, Sweden) before initiation of ICI therapy and were frozen until analysis. Total RNA was isolated from PBMCs using RNeasy Plus Universal Mini Kit (Qiagen, Hilden, Germany), according to the manufacturer’s instructions. RNA amounts and purities were measured using NanoDrop1000 (Thermo Scientific, Wilmington, Delaware, USA). RNA integrity was assessed using the RNA Nano 6000 Assay Kit of the Agilent Bioanalyzer 2100 system (Agilent Technologies, Santa Clara, California, USA). Construction of cDNA libraries followed by RNA sequencing was performed by Takara Bio (Shiga, Japan) as a contract analysis with SMART-Seq V.4 Ultra Low Input RNA Kit (Clontech, Palo Alto, California, USA) and a NovaSeq sequencing system (Illumina, San Diego, California, USA) according to the manufacturer’s instruction.

### Analysis of RNA sequencing data

After confirming the read quality with FastQC (https://www.bioinformatics.babraham.ac.uk/projects/fastqc/), sequence data were aligned to the human genome GRCh37 using STAR V.2.5.2a (https://github.com/alexdobin/STAR/releases/tag/2.5.2a), and the mapped read count of each sample was calculated using Python V.2.7-based HTseq. The obtained read count data for each PBMC sample were subjected to the Genomon 2 DNA analysis pipeline (https://github.com/Genomon-Project) to check sequencing qualities. The CIBERSORT algorithm (‘CIBERSORT’ R package, http://cibersort.stanford.edu) was applied to deconvolute the total transcriptome data for predicting the relative proportion of individual immune cell subtypes in the PBMCs of each patient based on an LM22 signature gene file.^11^ The ‘DESeq2’ Bioconductor R package V.1.28.1 (http://www.bioconductor.org/packages/release/bioc/vignettes/DESeq2/inst/doc/DESeq2.html) was carried out to normalize the process and identify the differentially expressed genes (DEGs) between two groups from the read count data of RNA sequencing. The threshold value to list DEGs was greater than 1.5-fold change with p<0.05. A list of amino acid metabolism-related genes (AAMGs), including those involved in amino acid metabolic pathways and amino acid transport, was generated from the Kyoto Encyclopedia of Genes and Genomes (KEGG, www.genome.jp/kegg) and the AmiGO V.2 browser (http://amigo.geneontology.org/amigo). Gene annotation analysis was performed for DEGs as a whole or DEGs listed as AAMGs (amino acid metabolism-related DEGs (DEG–AAMGs)) based on the Gene Ontology (GO, http://geneontology.org), KEGG, Reactome Pathway (REACTOME, https://reactome.org), and gene set enrichment analysis (GSEA, https://gsea-msigdb.org/gsea/index.jsp) databases. The ‘clusterProfiler’ V.3.16.1 and ‘ReactomePA’ V.1.32.0 R packages were used for enrichment data analysis and data visualization.^12^ P values were corrected for multiple comparisons by performing the R function p.adjust with the Benjamin and Hochberg method.

### Statistical analysis

The Cox proportional hazard model was used to analyze each patient’s characteristics, blood test results, PFAA and metabolite concentrations as an explanatory variable (univariate analysis) correlated with OS. Continuous variables were presented as mean and SD; they were compared between categorical groups using a t-test. Correlations between continuous variables were evaluated using Spearman’s rank correlation coefficient analysis. Univariate logistic regression was performed with the multivariate model score as the dependent variable and patient characteristics as independent variables to estimate OR and 95% CI. All statistical analyses were conducted using the R-language for statistical computing V.2.9.0 and V.4.0.2, and JMP V.15 (SAS Institute, Cary, North Carolina, USA).

## Results

### Patient characteristics and their association with OS

This study enrolled 53 patients treated with anti-PD-1 therapy. The overall response rate, median PFS, and median OS were 30.2%, 2.8 months (95% CI 1.4 to 4.9 months), and 7.6 months (95% CI 5.5 to 13.1 months), respectively. Overall, 19/53 patients (35.8%) developed treatment-related AEs, and 35 patients died during follow-up. The patients’ characteristics and blood test results are shown in table 1. Forty (75%) patients were male and 13 (25%) were never-smokers, and the mean age of all patients was 69.7 years. Seven (13%) patients had recurrence after concurrent chemoradiotherapy; 14 (26%) had recurrence after surgery; and 32 (60%) had stage IV. Twenty (38%) patients received PD-1 inhibitor as the first line, 22 (42%) as the second line, 7 (13%) as the third line, 2 (4%) as the fourth line, and 2 (4%) as the sixth or seventh line.

**Table 1:** Patient characteristics and their association with OS

Patient characteristics	Total (N=53)	Cox hazard model		
		HR	95% CI	P value
Age (years), mean (SD)	69.7 (8.5)	1.23	0.88 to 1.74	0.232
Sex, n (%)				
Female	13 (25)	1		
Male	40 (75)	0.92	0.44 to 1.92	0.826
Smoking, n (%)				
Former	40 (75)	1		
Never	13 (25)	1.46	0.71 to 3.01	0.318
Performance Status, n (%)				
0–1	38 (71)	1		
2–3	15 (29)	2.17	1.06 to 4.44	0.043
Stage, n (%)				
Stage III (recurrent after chemoradiotherapy)	7 (13)	1		
Recurrent after surgery	14 (26)	2.25	0.47 to 10.72	0.308
Stage IV	32 (60)	4.85	1.14 to 20.67	0.033
Histology, n (%)				
Non-squamous	38 (72)	1		
Squamous	15 (28)	1.38	0.65 to 2.92	0.410
Driver mutation, n (%)				
Wild Type	41 (77)	1		
EGFR (Epidermal growth factor receptor)	11 (21)	0.64	0.26 to 1.56	0.306
ALK (Anaplastic lymphoma kinase)	1 (2)	(EGFR or ALK/WT)		
Tumor PD-L1 expression, n (%)				
0%–49%	21 (45)			
50%–100%	26 (55)	1.23	0.60 to 2.51	0.232
Treatment line, n (%)				
1st line	20 (38)	1		
2nd line	22 (42)	0.76	0.36 to 1.60	0.471
3rd line	7 (13)	0.75	0.30 to 1.88	0.540
4th line	2 (4)	(3rd line or later/1st line)		
6th and 7th lines	2 (4)			
PD-1 blocker, n (%)				
Nivolumab	25 (47)	1		
Pembrolizumab	28 (53)	1.37	0.70 to 2.66	0.358
Blood test, mean (SD)				
Albumin (g/dL)	3.42 (0.61)	0.57	0.38 to 0.85	0.005
LDH (lactate dehydrogenase, U/L)	2634 (118)	1.13	0.81 to 1.49	0.448
White blood cell (/μL)	7513 (3357)	1.35	0.98 to 1.75	0.062
Lymphocyte (/μL)	1354 (615)	0.58	0.38 to 0.86	0.006
Neutrophil (/μL)	5476 (2969)	1.50	1.10 to 1.97	0.011
Eosinophil (/μL)	176 (178)	0.80	0.52 to 1.15	0.248
Monocyte (/μL)	477 (223)	1.49	1.05 to 2.02	0.027
Neutrophil:lymphocyte ratio	4.94 (3.56)	1.72	1.27 to 2.25	<0.001

Categorical variables are shown as the distribution of corresponding patient numbers. Continuous variables are shown as mean and SD values.

Univariate analysis was conducted using the Cox proportional hazard model for OS. HR, 95% CI.

ALK, anaplastic lymphoma kinase; LDH, lactate dehydrogenase; OS, overall survival; PS, performance status; WT, wild type.

On the univariate analysis, performance status and tumor stage showed significant correlations with OS (p=0.043 and 0.033, respectively). In the blood test results, albumin levels, lymphocyte counts, neutrophil counts, monocyte counts, and neutrophil:lymphocyte ratios (NLRs) before ICI therapy were significantly correlated with OS (p=0.005, 0.006, 0.011, 0.027, and <0.001, respectively). Regarding the treatment lines, there were no significant differences in OS between the first line and the second line or the third line or later in patients with both low (0%–49%) and high (50%–100%) PD-L1 expression levels (online supplemental figure 1A). Of the 12 patients positive for driver gene mutations, most (92%) patients receive PD-1 inhibitors as the third-line or later after molecular targeted therapy and/or chemotherapeutic agents had failed, but half of the enrolled patients showed good antitumor responses (Partial Response, n=6) in this study (online supplemental figure 1).

10.1136/jitc-2021-004420.supp1 Supplementary data

### Association between PFAA and metabolite concentrations and OS

Higher concentrations of seven PFAAs, including glycine, histidine, threonine, alanine, citrulline, arginine, and tryptophan, before ICI therapy were significantly correlated with better OS on Cox proportional hazard analysis (p=0.049, 0.009, 0.015, 0.006, 0.018,<0.001, and 0.005, respectively) (table 2). In contrast, lower concentrations of three tryptophan-related metabolites, including 3h-kynurenine, anthranilic acid, and quinolinic acid, and neopterin before ICI therapy were significantly correlated with better OS (p=0.002, 0.007, 0.049, and 0.014, respectively). The concentrations of serine, histidine, threonine, alanine, arginine, tryptophan, anthranilic acid, xanthurenic acid, and neopterin stratified by the median value also showed significant differences in OS when analyzed by log-rank testing (online supplemental figure 2).

**Table 2:** Association between PFAA and metabolite concentrations before ICI therapy and OS

PFAA/metabolite (μM)	Mean (SD)	Cox hazard model		
		HR	95% CI	P value
Glutamic acid	50.5 (22.2)	1.03	0.71 to 1.45	0.863
Serine	101.6 (27.4)	0.72	0.50 to 1.03	0.075
Asparagine	41.9 (10.8)	0.73	0.47 to 1.06	0.107
Glycine	207.4 (64.5)	0.69	0.45 to 1.00	0.049
Glutamine	543.4 (101.6)	0.77	0.51 to 1.16	0.213
Histidine	65.0 (23.0)	0.52	0.30 to 0.86	0.009
Threonine	107.4 (41.8)	0.58	0.34 to 0.91	0.015
Alanine	305.2 (110.6)	0.60	0.41 to 0.87	0.006
Citrulline	30.4 (13.1)	0.61	0.39 to 0.92	0.018
Arginine	74.1 (20.3)	0.42	0.27 to 0.64	<0.001
Proline	151.3 (63.3)	0.80	0.53 to 1.11	0.199
α-Amino butyric acid	17.5 (6.0)	0.99	0.70 to 1.39	0.977
Tyrosine	63.5 (17.4)	0.79	0.52 to 1.14	0.234
Valine	210.5 (63.7)	0.78	0.52 to 1.12	0.193
Methionine	22.1 (7.5)	0.75	0.46 to 1.12	0.183
Ornithine	64.9 (13.4)	0.72	0.46 to 1.10	0.126
Lysine	175.7 (49.1)	0.70	0.47 to 1.03	0.069
Isoleucine	69.2 (29.7)	0.90	0.56 to 1.22	0.566
Leucine	120.8 (47.7)	0.87	0.55 to 1.22	0.471
Phenylalanine	62.1 (13.3)	1.23	0.83 to 1.82	0.294
Tryptophan	46.8 (17.8)	0.53	0.34 to 0.83	0.005
3h-kynurenine	0.069 (0.050)	1.85	1.27 to 2.60	0.002
3h-anthranilic acid	0.047 (0.036)	1.37	0.86 to 1.96	0.166
5h-indol-3-acetic acid	0.070 (0.062)	1.02	0.68 to 1.33	0.923
5h-tryptophan	0.005 (0.009)	1.38	0.99 to 1.74	0.054
Anthranilic acid	0.019 (0.016)	1.47	1.13 to 1.82	0.007
Indol-3-acetic acid	1.996 (1.771)	0.81	0.51 to 1.12	0.236
Indol-3-lactic acid	0.862 (0.551)	0.96	0.67 to 1.28	0.810
Kynurenic acid	0.049 (0.027)	1.22	0.83 to 1.73	0.293
Kynurenine	2.448 (0.716)	1.37	0.96 to 1.95	0.083
Picolinic acid	0.054 (0.033)	1.18	0.82 to 1.63	0.358
Quinolinic acid	0.730 (0.515)	1.34	1.00 to 1.69	0.049
Serotonin	0.159 (0.152)	1.09	0.70 to 1.55	0.680
Xanthurenic acid	0.011 (0.012)	0.91	0.60 to 1.29	0.572
Neopterin	0.010 (0.009)	1.50	1.10 to 1.92	0.014
N′-formyl-kynurenine	0.018 (0.014)	0.82	0.46 to 1.14	0.295

The concentrations of PFAAs and metabolites are shown as mean and SD values.

Univariate analysis of PFAAs and metabolites before ICI therapy was conducted using the Cox proportional hazard model for OS. HR, 95% CI and P values are shown.

ICI, immune checkpoint inhibitor; OS, overall survival; PFAA, plasma-free amino acid.

### Development of a multivariate model for stratifying prognosis in ICI-treated patients

The PFAA and metabolite variables that showed a trend of correlation with OS in the univariate analysis (p<0.1) were selected as variables in the multivariate model. These included the following 15 PFAAs and metabolites: serine, glycine, histidine, threonine, alanine, citrulline, arginine, lysine, tryptophan, 3h-kynurenine, 5h-tryptophan, anthranilic acid, kynurenine, quinolinic acid, and neopterin. A multivariate model was optimized through a stepwise method to minimize AIC. We created the best multivariate model comprising three PFAAs and one metabolite, namely, serine, glycine, arginine, and quinolinic acid as variables, expressed by the combination of each variable and the corresponding coefficient, F=a+b*serine+c*glycine+d*arginine+e*quinolinic acid. The discrimination performance of this model was as follows: C-index=0.775 (no validation), C-index=0.742 (the combination of variables was validated with LOOCV), and Cox HR=3.23 (95% CI 2.04 to 5.26).

**Figure 1:**
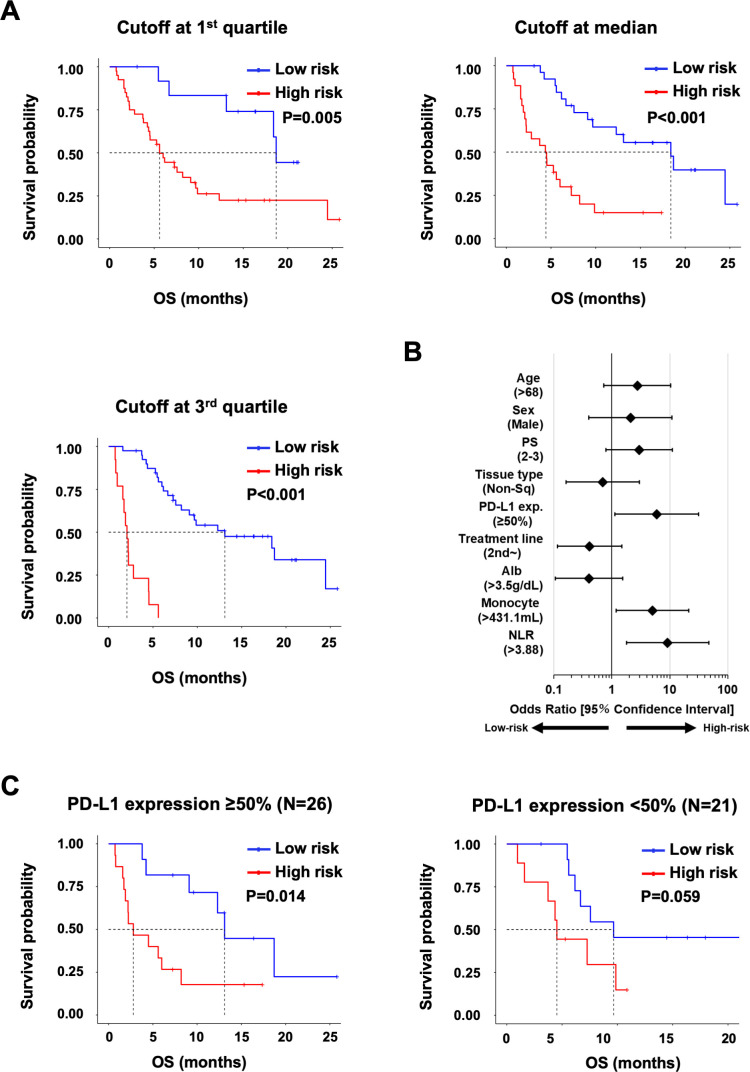
Multivariate model for prognosis of patients treated with immune checkpoint inhibitor. (A) Kaplan-Meier analysis for OS in the subgroups stratified by the multivariate model. The cut-off values between the high-risk and the low-risk groups at the following three points: first quartile value, median value and third quartile value. Cox HR (95% CI) and p value (log-rank test) are shown. (B) Univariate regression analysis for the association of patient characteristics with the multivariate model. The cut-off value of the multivariate model was set at the third quartile score. Continuous prognostic factors were divided at the median value. ORs about tumor stage and mutation were not determined because all patients with stage III (recurrent after chemoradiotherapy) or Epidermal growth factor receptor mutations were stratified into the high-risk group. (C) Kaplan-Meier analysis for OS in the subgroups stratified by the multivariate model in the high (≥50%, n=26) or low (<50%, n=21) PD-L1 expression group. The cut-off values between the high-risk and low-risk groups were set at the median value. Cox HR (95% CI) and p value (log-rank test) are shown. NLR, neutrophil:lymphocyte ratio; OS, overall survival.

Figure 1A shows the results of discrimination with this multivariate model at three cut-off values: (1) first quartile, (2) median, and (3) third quartile. In this multivariate model, the higher the score, the worse the prognosis; thus, we defined high-risk groups when the score of the multivariate model was higher than the cut-off value. When the cut-off value of the third quartile was selected, the model could discriminate patients to the high-risk group (MST=2.1, 95% CI 0.97 to 2.8) and low-risk group (MST=13.1, 95% CI 7.6 to 24.5) with an HR of 15.79 (95% CI 6.15 to 40.52). We performed univariate regression analysis to investigate which factors were associated with the multivariate model and found that this model was significantly correlated with monocyte count and NLR (figure 1B). Furthermore, we evaluated the independency of prognostic factors, such as tumor stage, performance status, tumor PD-L1 expression, albumin level, monocyte count, NLR, and the multivariate model (table 3). Since neutrophil and lymphocyte counts had a direct inter-relation with NLR, they were not included in this analysis. As a result, performance status and the multivariate model were selected as significantly independent factors (p=0.008 and p=0.005, respectively).

**Table 3:** Multivariate analysis of prognostic factors for overall survival

Prognostic factors	Cox hazard model		
	HR	95% CI	P value
Tumor stage (recurrent after surgery and stage VI/recurrent-after chemoradiotherapy)	5.00	0.64 to 39.06	0.125
Performance status (2–3/0–1)	3.18	1.35 to 7.51	0.008
PD-L1 expression (50%–100%/0%–49%)	1.24	0.56 to 2.78	0.596
Monocyte (median)	0.64	0.29 to 1.40	0.264
Neutrophil:lymphocyte ratio (median)	2.17	0.75 to 6.25	0.151
Albumin (median)	1.59	0.68 to 3.69	0.283
Multivariate model (median)	3.55	1.47 to 8.59	0.005

Each prognostic factor for overall survival was evaluated by multivariate Cox proportional hazard analysis.

We further evaluated the multivariate prediction model in the subgroups stratified by PD-L1 expression levels in tumors (figure 1C). When the cut-off value of the multivariate model was set in the median score, the high-risk group showed worse OS in higher (≥50%, n=26) PD-L1 expression subgroups (log-rank test, p=0.014).

### Total transcriptome analyses in PBMCs obtained from high-risk and low-risk patients

We investigated the relationships between PFAAs/metabolites and transcriptome in PBMCs to elucidate potential molecular mechanisms of amino acid metabolism. Among the 53 patients analyzed previously, PBMCs from 36 patients before ICI treatment were available for total transcriptome analyses by RNA sequencing. We separated the patients into the low-risk (n=30) and high-risk (n=6) groups according to the cut-off at the third quartile on the established multivariate model. Online supplemental tables 1 and 2 show the information of the 36 patients analyzed, including patient characteristics, blood test results, and concentrations of PFAAs and tryptophan-related metabolites, and neither of them in these 36 patients had significant differences with those in the original 53 patients. The patients in the high-risk group had a significantly worse OS after ICI therapy (online supplemental figure 3A), and MST of both high-risk (2.5 months) and low-risk (13.6 months) groups showed no significant differences with those in the original 53 patients (2.1 and 13.1 months, respectively). The risk scores by the multivariate model with PFAAs/metabolites were compared using a Mann-Whitney-Wilcoxon test between the subgroups classified by the antitumor responses (RECIST criteria). The distribution of risk scores in the multivariate model showed significant differences between the tumor response PD and PR groups, similar to the original 53 patients (online supplemental figure 3B). These data suggested that the 36 patients, whose PBMCs were evaluated by RNA sequencing, retained the same clinical characteristics as the original 53 patients.

10.1136/jitc-2021-004420.supp2 Supplementary data

**Figure 2:**
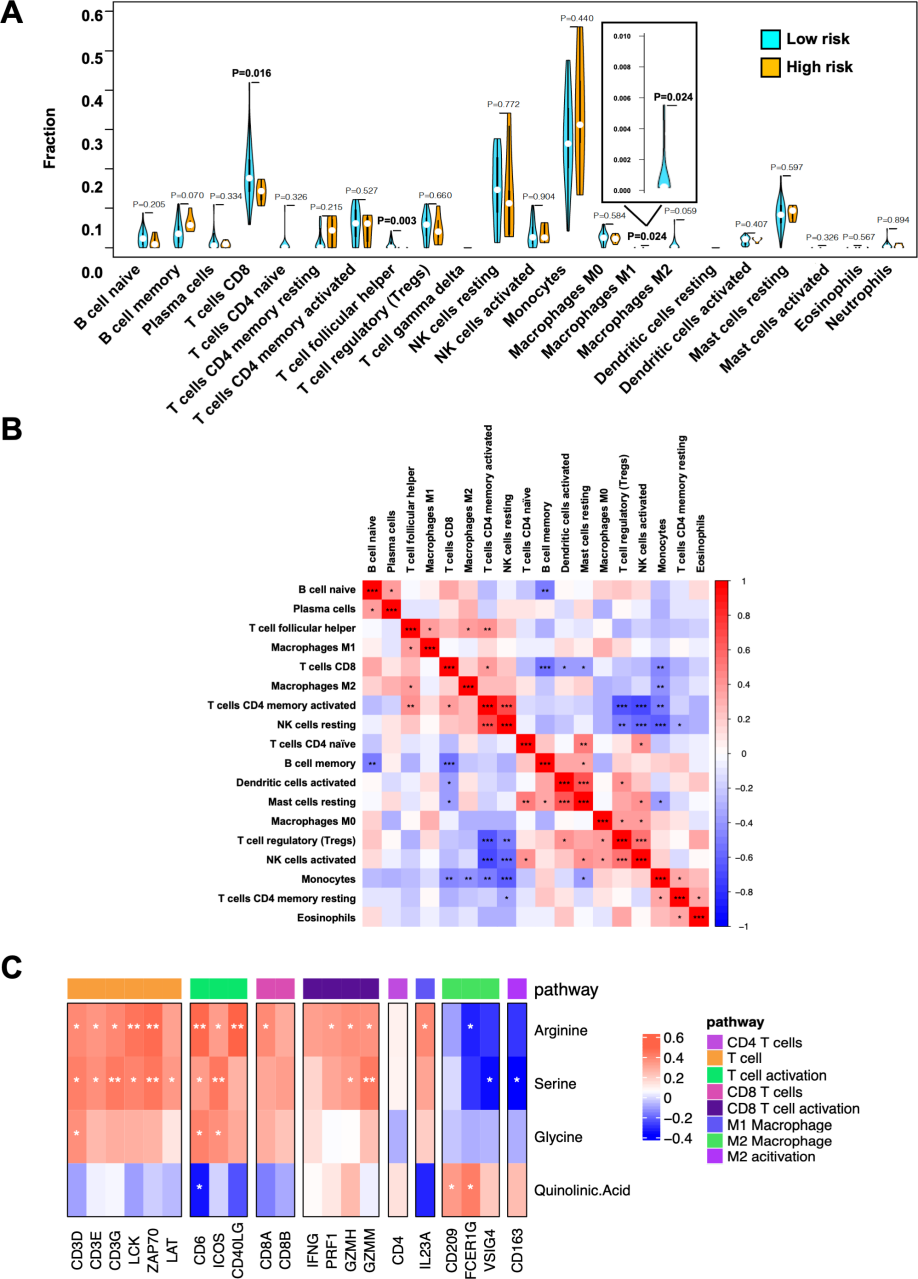
Immune cell constitution of PBMCs in the high-risk and low-risk groups. (A) Violin plots depicting immune cell subtypes in PBMCs from the high-risk and low-risk groups by the multivariate model. Fractions of immune cells were deconvoluted by the CIBERSORT algorithm from RNA-seq data in PBMCs and compared between the high-risk and low-risk groups using Student’s t-test. Blue, low-risk group; orange, high-risk group. (B) Heatmap of Spearman’s correlations among immune cell subtypes. Red, positive correlation; blue, negative correlation; white, no correlation. (C) Correlations between immune-related gene expression in PBMCs and PFAAs/tryptophan metabolites. Heatmap of Spearman’s correlations between gene expression levels of immune-related genes and concentrations of 4 PFAAs/metabolite selected in the multivariate model (arginine, serine, glycine, and quinolinic acid) are shown. The genes were grouped by immune pathways. Red, positive correlation; blue, negative correlation; white, no correlation. NK, natural killer; PBMC, peripheral blood mononuclear cell.

The immune cell constitution of PBMCs obtained from the high-risk and low-risk patients was deduced by deconvoluting the bulk RNA sequencing data with the CIBERSORT algorithm. Among the deduced 22 immune cell subtypes, the proportions of CD8^+^ T cells, follicular helper T cells, and M1 macrophages were significantly abundant in the low-risk group (p=0.016, 0.003, and 0.024, respectively), as shown in figure 2A. The relative abundance of CD8^+^ T cells in the PBMCs was significantly positively correlated with CD4^+^ memory-activated T cells and significantly negatively correlated with memory B cells, monocytes, resting mast cells, and activated dendritic cells, with a p value of <0.05 (figure 2B).

We also examined the correlations between the concentrations of PFAAs/metabolite selected in the multivariate model (arginine, serine, glycine, and quinolinic acid) and the expressions of immune-related genes, especially those of T cell-related genes. As shown in figure 2C, the plasma concentrations of arginine and serine were positively correlated with the expressions of genes of TCR-mediated signaling pathways, such as *CD3D*, *CD3E*, *CD3G*, *LCK*, *ZAP70*, and *LAT*, and T-cell activation, such as *CD6*, *ICOS*, and *CD40 Ligand*, whereas the quinolinic acid concentration tended to be negatively correlated with them. Of note, the arginine and serine concentrations also tended to be positively correlated with the expressions of genes of CD8 T cells and their activation, such as *CD8A*, *CD8B*, *IFN-γ*, *Perforin*, and *Granzymes*, but not with CD4-related genes. Furthermore, arginine and serine tended to be negatively correlated with genes related to M2 macrophages, such as *CD209*, *FCER1G*, *VSIG4*, and *CD163*, but quinolinic acid tended to be positively correlated with them.

### Identification and characterization of DEGs between the high-risk and low-risk patients

Through analysis of RNA sequencing data of PBMCs, 492 DEGs were identified between the low-risk and high-risk groups. Top 10 GO terms obtained by GO term enrichment analysis for the identified DEGs included the terms ‘T cell activation’, ‘Lymphocyte differentiation’, ‘Nuclear division’, ‘Regulation of T cell activation’, ‘Mitotic nuclear division’, ‘Regulation of leukocyte cell-cell adhesion’, ‘T cell differentiation’, ‘Regulation of nuclear division’, ‘Regulation of mitotic nuclear division’, and ‘T cell proliferation’ (online supplemental figure 4A). In addition, immune-related GO terms, such as ‘Regulation of lymphocyte activation’, ‘Positive regulation of cell cycle’, ‘Macrophage activation’, ‘Regulation of interferon-gamma production’, ‘Regulation of inflammatory response to antigenic stimulus’, and ‘Regulation of dendritic cell differentiation’ were also obtained by this analysis (p<0.05) (online supplemental table 3).

Analysis of enrichment pathways in DEGs by KEGG identified ‘*Staphylococcus aureus* infection’, ‘complement and coagulation cascades’, ‘pertussis’, and ‘cell cycle’ as upregulated pathways in the high-risk group, whereas ‘NF-kappa B signaling pathway’, ‘T cell receptor signaling pathway’, ‘hematopoietic cell lineage’, and ‘primary immunodeficiency’ were downregulated (p<0.05) (online supplemental figure 4B and online supplemental table 4). We further evaluated the RNA sequencing data across different pathways by GSEA between the high-risk and low-risk groups. ‘Complement cascade’, ‘regulation of TLR by endogenous ligand’, ‘interferon alpha beta signaling’, ‘cell cycle checkpoints’, ‘biological oxidations’ and ‘Toll-like receptor TLR1:TLR2 cascade’ showed a significantly positive enrichment in the low-risk group (p<0.05). In contrast, ‘cell–cell junction organization’ and ‘antigen activated B-cell receptor leading to generation of second messengers’ were positively enriched in the high-risk group (p<0.05) (online supplemental figure 4C).

### Analysis of DEG–AAMGs

**Figure 3:**
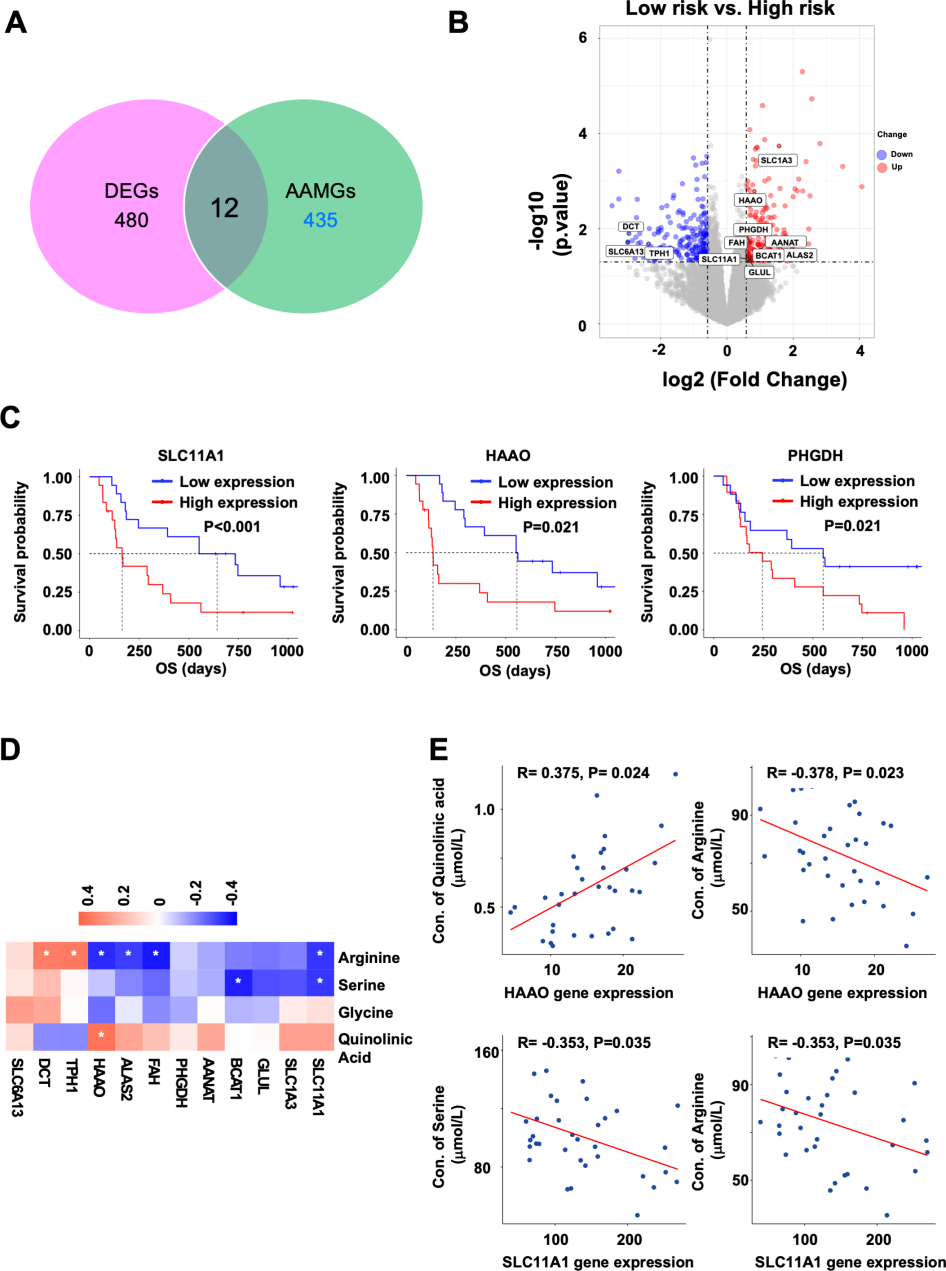
Identification of AAMGs differentially expressed between the high-risk and low-risk groups. (A) Venn plot of AAMGs among the DEGs. (B) Volcano plot of DEGs. AAMGs were labeled in the plot with gene names. Red dots, upregulated genes; blue dots: downregulated genes; gray dots, stable genes. (C) Kaplan-Meier estimates of OS in the subgroups stratified by RNA expression level of three AAMGs, *SLC11A1*, *HAAO*, and *PHGDH*, in PBMCs. Cut-off values between the high and low groups were set at the median of gene expression levels. P values (log-rank test) are shown. (D) Correlations between 12 DEG–AAMGs in PBMCs and PFAAs/tryptophan metabolites. Heatmap of Spearman’s correlations between expression levels of 12 DEG–AAMGs and concentrations of 4 PFAAs/metabolites selected in the multivariate model (arginine, serine, glycine, and quinolinic acid) are shown. Red, positive correlation; blue, negative correlation; white, no correlation. (E) Scatter plots of the *HAAO* gene expression in PBMCs versus concentrations of quinolinic acid or arginine (left half). Scatter plots of the *SLC11A1* gene expression in PBMCs verus concentrations of serine or arginine (right half). The correlations were evaluated by Spearman’s rank correlation coefficient analysis. R indicates correlation coefficient. amino acid metabolism-related gene; DEG, differentially expressed gene; *HAAO*, *3-Hydroxy Anthranilic Acid Dioxygenase*; OS, overall survival; PFAA, plasma-free amino acid.

Among 492 DEGs identified between the low-risk and high-risk groups, 12 were amino acid metabolism-related genes (DEG–AAMGs), as shown in figure 3A. The high risk group showed upregulation of 9 DEG–AAMGs, including *SLC1A3*, *3-Hydroxy Anthranilic Acid Dioxygenase* (*HAAO*), *PHGDH*, *AANAT*, *ALAS2*, *FAH*, *BCAT1*, *SLC11A1*, and *Glutamate-Ammonia Ligase* (*GLUL*), and dowregulation of 3 DEG–AAMGs, including *DCT*, *SLC6A13*, and *TPH1* (figure 3B). Annotation of these DEG–AMGs by KEGG enrichment pathways identified ‘biosynthesis of amino acids’, ‘glysine, serine, and threonine metabolism’, ‘biosynthesis of cofactors’, ‘cysteine and methionine metabolism’, ‘glutamatergic synapse’, ‘arginine biosynthesis’, ‘pantothenate and CoA biosynthsis’, ‘tryptophan metabolism’, ‘2-oxocarboxylic acid metabolism’, and ‘nitrogen metabolism’ as upregulated pathways in the high-risk group. In contrast, ‘folate biosynthesis’, ‘tyrosine metabolism’, ‘tryptophan metabolism’, ‘synaptic vesicle cycle’, ‘GABAergic synapse’, ‘melanogenesis’, ‘serotonergic synapse’, and ‘tryptophan metabolism’pathways were downregulated in the high-risk group (p<0.05) (online supplemental figure 5).

### Association between AAMG expression in PBMCs and patient prognosis

We further examined the relationships between AAMG expression in PBMCs and OS in the 36 patients treated with ICI. Expression levels of 20 AAMG mRNA were significantly correlated with the OS; upregulation of 3 AAMGs, *VAMP2*, *PER2*, and *ACY1*, was positively correlated with OS, whereas upregulation of 17 AAMGs, *UNC13B*, *SH3BP4*, *SFXN3*, *SFXN2*, *ASL*, *ASH1L*, *ACAA2*, *PLOD3*, *ACAT1*, *PSAT1*, *ACADS*, *ALDH3B1*, *ALDH2* and *HMGCL, SLC11A1*, *HAAO*, and *PHGDH*, was negatively correlated with OS (log-rank test, p<0.05) (online supplemental figure 6 and figure 3C). Notably, *SLC11A1*, *HAAO*, and *PHGDH* were also selected as DEG–AAMGs in the preceding analysis.

We also performed Spearman’s correlation coefficient analysis to investigate the relationships between the PFAAs/metabolite selected as the components of the multivariate model and the expressions of DEG–AAMGs. Heatmap of Spearman’s correlations between the expression levels of 12 DEG–AAMGs and concentrations of 4 PFAAs/metabolite, including arginine, serine, glycine, and quinolinic acid, are shown in figure 3D. Of note, among the three DEG–AAMGs with survival significance, the *HAAO* expression was positively correlated with the plasma quinolinic acid (R=0.375, p=0.024) and negatively correlated with the plasma arginine (R=−0.378, p=0.023), whereas the *SLC11A1* expression was negatively correlated with the plasma arginine (R=−0.353, p=0.035) and serine (R=−0.353, p=0.035) (figure 3E). However, the expression of *PHGDH* gene was not significantly correlated with the plasma glycine (R=−0.183, p=0.285) or serine (R=−0.123 p=0.474) (data not shown).

## Discussion

In the present study, we showed that the optimized multivariate model with PFAAs and metabolites in plasma can stratify the prognosis in patients with NSCLC treated with ICI. Notably, this multivariate model was a significantly independent factor for OS. In addition, it was useful for stratifying the prognosis in the subgroup with higher PD-L1 expression. Therefore, our finding might help develop a novel biomarker for ICI treatment, which could have different values from current biomarkers, such as PD-L1 expression.

Amino acid metabolism in the tumor microenvironment (TME) plays key roles in tumor development and progression. Tumor cells often exclusively consume regional nutrients, such as amino acids, for their survival and compete for them with other surrounding cells, such as antitumor immune cells. It may thus be noteworthy that the plasma PFAAs and metabolites before ICI therapy were significantly correlated with OS. The best multivariate model for stratifying the prognosis of patients treated with ICI was composed of four PFAAs/metabolite, namely, serine, glycine, arginine, and quinolinic acid. Among these amino acids, serine and glycine are known to form one-carbon pathway involved in the anabolism of multiple macromolecular substances, such as proteins, nucleic acids, lipids and other biological molecules, to support tumor growth.^13^ Arginine is known as substrate of nitric oxide and might regulate proliferation, cell death and angiogenesis in cancer cells.^14 15^ Of note, these amino acids are also important for metabolism of immune cells and might substantially contribute to antitumor immunity. For example, extracellular serine was reported to be one of the key immunometabolites that modulate adaptive immunity by controlling T-cell proliferative capacity.^16^ Arginine is also widely recognized as a major contributor to metabolic reprogramming through control of polyamine synthesis for immune cell proliferation and nitric oxidase synthesis for cytotoxicity. In addition, enhanced arginine uptake from extracellular sources induces metabolic changes in T cells, including a shift from glycolysis to oxidative phosphorylation in activated T cells, and promotes generation of central memory-like cells with stronger antitumor activity and longer survival capacity.^17^ Furthermore, infiltrating myeloid-derived suppressor cells expressing arginase were reported to suppress the efficacy of ICI therapy.^18 19^ Interestingly, in the present study, the concentrations of plasma arginine and serine were significantly correlated with the expressions of T cell-related genes in PBMCs. Considering their critical roles in T-cell survival and activation,^16 17^ it might be likely that they regulate T-cell functions directly.

The metabolism of the tryptophan–kynurenine pathway might not directly affect tumor cells but may promote tumor progression through modulation of the immunosuppressive microenvironment via multiple mechanisms. For example, tryptophan-metabolizing enzymes IDO (indoleamine 2,3-dioxygenase) /TDO (tryptophan 2, 3-dioxygenase) were reported to affect antitumor immunity in various tumors.^20 21^ In the present study, our Cox proportional hazard analysis demonstrated that decrease in tryptophan and increase in kynurenine-derived metabolites, including 3h-kynurenine, anthranilic acid, and quinolinic acid, were significantly correlated with poor OS, possibly due to impaired antitumor immunity. In addition, the concentration of quinolinic acid tended to be negatively correlated with the expressions of T cell-related genes in PBMCs. Since quinolinic acid is a downstream catabolite of tryptophan, which is known to play a critical role in T-cell activation, it might be possible that tryptophan catabolism, which increases quinolinic acid, is associated with T-cell suppression and dysfunction. Moreover, since consumption of tryptophan and accumulation of its metabolites are known to activate specific genes, such as AhR and GCN2, which drive a tolerogenic macrophage phenotype, indicating that they might directly regulate myeloid cell functions.^22^ Consistent with our results, similar metabolic features on tryptophan metabolites were reported to correlate with the prognosis of patients treated with ICI.^23 24^

Antitumor immune cells are suggested to continuously circulate between the TME and the peripheral blood, as illustrated in online supplemental figure 7. Thus, the immune cells in the peripheral blood may reflect the composition, activity, and metabolic characteristics of those within the TME.^25 26^ Indeed, in the present study, when the composition of immune cells was examined by deconvoluting whole transcriptomic data of PBMCs with the CIBERSORT algorithm, CD8^+^ T cells that have prognostic relevance for tumor immunotherapy^27 28^ were significantly increased in the low-risk group according to the multivariate model. In addition, the GO term enrichment analysis of the PBMC transcriptome demonstrated that DEGs identified between the low-risk and high-risk groups were significantly associated with T-cell proliferation, differentiation, and activation. Furthermore, the KEGG pathway annotation and GSEA analysis of reactome pathways showed that the pathways associated with proinflammatory immune responses were significantly enriched in the low-risk group. Taken together, these results suggested that the PBMCs’ transcriptome in the low-risk group might reflect the so-called ‘hot tumor’ characteristics in TME.^29^

Interestingly, the expressions of 2 DEG–AAMGs with prognostic significance, *HAAO* and *SLC11A1*, showed weak but significant correlations with some of PFAA/metabolite concentrations in the present study. The expression of *HAAO* was positively correlated with the plasma quinolinic acid concentration. *HAAO* is an enzyme involved in the metabolism of tryptophan–kynurenine pathway and reported to catalyze the synthesis of quinolinic acid.^30^ Therefore, higher *HAAO* expression in PBMCs might directly contribute to higher plasma concentration of quinolinic acid through catabolism of tryptophan. In contrast, the expression of *SLC11A1* (*NRAMP1*) encoding a proton-coupled metal ion transporter was negatively correlated with the plasma arginine concentration. *SLC11A1* is expressed in the lysosomal compartment of macrophages and translocates to the phagolysosome membrane when activated. Previous reports showed that *SLC11A1* increases the expression of *iNOS*, an arginine metabolizing enzyme, and eliminates intracellular pathogens.^31^ Thus, it might be reasonable that higher *SLC11A1* expression in PBMCs directly contributes to reduction of plasma arginine concentration. Based on these results, high expression of *HAAO* and *SLC11A1* may lead to decrease in tryptophan and arginine, respectively, resulting in immune cell suppression in the high-risk group in our multivariate model. In contrast, another DEG–AAMG with prognostic significance, *Phosphoglycerate Dehydrogenase* (*PHGDH*), is a key enzyme for de novo serine/glycine synthesis, but the *PHGDH* expression level in PBMCs was not significantly correlated with the plasma glycine or serine in this study. Elevated expression of *PHGDH* is reported to be associated with tumor development in various tumors^32^ and poor prognosis in patients with cancers, including NSCLC.^33^ It was also reported to be involved in polarization and proliferation of M2 macrophage.^34^ Since deficiency of serine/glycine was reported to increase the enzmes for serine/glycine synthesis, including *PHGDH*,^35^ such negative feedback mechanisms might prevent direct correlation between the *PHGDH* expression and serine/glycine concentrations.

In this study, the following nine genes were further identified as DEG–AAMGs (figure 3B). *BCAT1* is an enzyme that converts branched-chain amino acids into the corresponding branched-chain α-keto acids and generates glutamate, and was reported to be associated with tumor growth and progression. BCAT1 was also shown to control metabolic reprogramming in activated human macrophages.^36^ *GLUL* is an enzyme for de novo synthesis of glutamine by catalyzing the ATP-dependent condensation of glutamate with ammonia. It was reported to bias macrophages towards an M1-like phenotype and inhibit tumor metastasis.^37^ *SLC1A3* is a glutamate/aspartate transporter that uses aspartate to support cells in the absence of extracellular glutamine. Since *SLC1A3* expression promotes the synthesis of glutamate and glutamine, tumor cells with high levels of *SLC1A3* expression were resistant to glutamine starvation and SLC1A3 deprivation retarded the tumor growth.^38^ *ALAS2* is a mitochondrial enzyme which uses glycine and succinyl-CoA to form 5-aminolevulinic acid (ALA), a crucial precursor in heme synthesis.^39^ *SLC6A13* belongs to the neurotransmitter transporter family and promotes ALA-induced accumulation of protoporphyrin IX and photodamage through ALA uptake by cancer cells.^40^ *AANAT* and *TPH1* are involved in tryptophan metabolism for melatonin and serotonin synthesis. *AANAT* modulates the phagocytic activity,^41^ whereas *TPH1* regulates immunological tolerance.^42^ *FAH*^43^ and *DCT*^44^ are mainly involved in tyrosine metabolism; *FAH* catalyzes the final step of tyrosine degradation, whereas *DCT* is a zinc enzyme associated with the melanogenic process of flavonoids. Since the roles of most of these genes, including *SLC1A3*, *ALAS2*, *SLC6A13*, *AANAT*, *TPH1*, *FAH*, *and DCT*, in immune cells, have not been fully elucidated, functional mechanisms of these genes remain to be further investigated.

In conclusion, our findings suggested that the profiling of PFAAs and metabolites in plasma might be useful for stratifying patients who will benefit from ICI treatment. Nevertheless, the present study had several limitations. The investigation was conducted in a single-center cohort with a relatively small number of patients who receive anti-PD-1 antibody without concurrent chemotherapy. However, the next phase of clinical trial has been ongoing as a larger multicenter cohort study to further optimize the multivariate model with PFAAs and metabolites in patients with NSCSC who receive combined treatment with anti-PD-1 antibody and chemotherapy (jRCT1031190196).

## Data Availability

Data are available upon reasonable request. Data are available from the corresponding authors upon reasonable demand, with the exception of absolute values of PFAA and metabolite analysis.
