# Supplementary material for: Clinical significance of plasma-free amino acids and tryptophan metabolites in patients with non-small cell lung cancer receiving PD-1 inhibitor: a pilot cohort study for developing a prognostic multivariate model
Source: J Immunother Cancer. 2022 May 11;10(5):e004420. doi: 10.1136/jitc-2021-004420 (PMC9109096; doi:10.1136/jitc-2021-004420)

Supplementary Figure 1

A

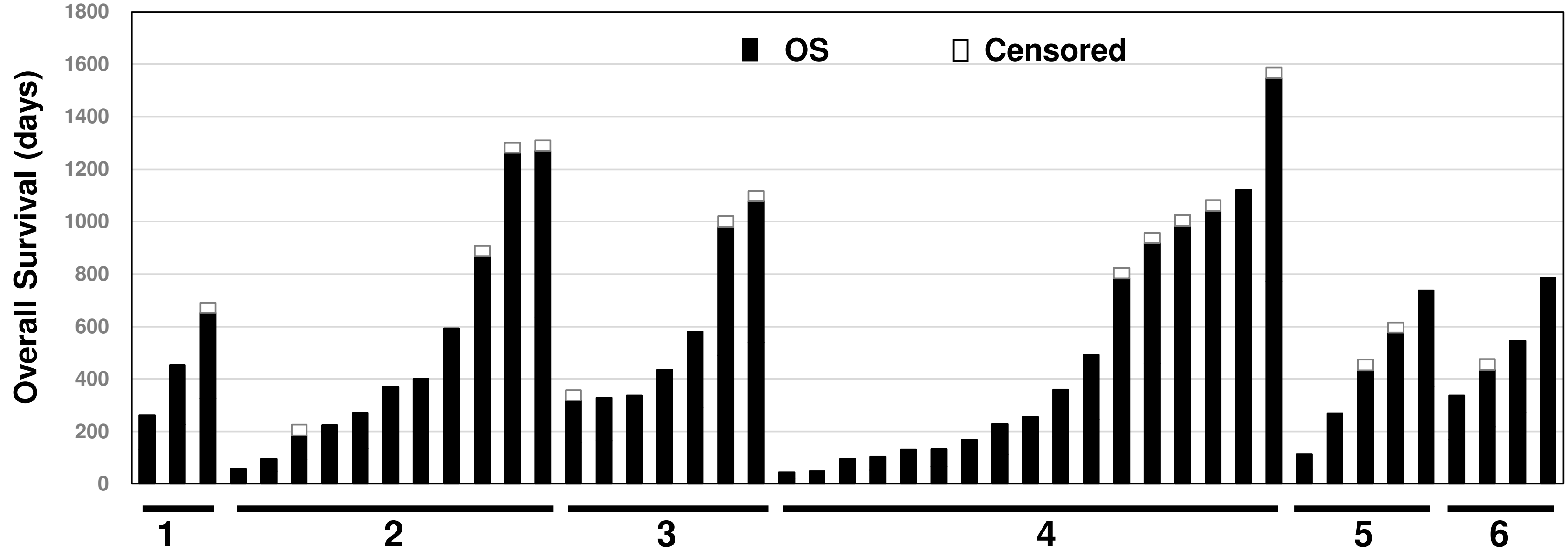

1: PD-L1 0-49% 1st line
2: PD-L1 0-49% 2nd line
3: PD-L1 0-49% 3rd line or later
4: PD-L1 50-100% 1st line
5: PD-L1 50-100% 2nd line
6: PD-L1 50-100% 3rd line or later

PD-L1 expression	Treatment Line	N	COX Hazard model		
			HR	95%CI	P value
0-49 %	1st	3	1.00	-	-
	2nd	11	1.21	0.25-5.85	0.813
	3rd or later	7	0.87	0.16-4.76	0.871
50-100 %	1st	17	1.00	-	-
	2nd	5	0.86	0.24-3.14	0.865
	3rd or later	4	0.86	0.24-3.14	0.817

Supplementary Figure 1

B

Pt	Age	Sex	Histology	Driver	Response	OS(days)	Censored	1st line	2nd line	3rd line	4th line	5th line	6th line	7th line
1	84	F	non-Sq	ALK	PR	168	1	Alectinib	Ceritinib	Pembrolizumab				
2	64	F	non-Sq	EGFR	SD	290	1	Gefitinib	CDDP+PEM	Erlotinib	Afatinib	Osimertinib	Afatinib	Pembrolizumab
3	57	F	non-Sq	EGFR	PD	273	1	CDDP+PEM	Erlotinib	Pembrolizumab				
4	77	F	non-Sq	EGFR	PD	218	1	CDDP+VNR	Gefitinib	CBDCA +PEM+Bev	DTX	Erlotinib	Nivolumab	
5	67	M	non-Sq	EGFR	PR	620	0	Erlotinib +Ramcirumab	Nivolumab					
6	67	M	non-Sq	EGFR	PR	539	0	Erlotinib +Ramcirumab	CDDP+PEM	Nivolumab				
7	78	M	non-Sq	EGFR	PD	164	1	CBDCA+PTX	Afatinib	Osimertinib	Pembrolizumab			
8	83	F	non-Sq	EGFR	PD	393	1	Afatinib	CBDCA +PEM	Nivolumab				
9	81	M	non-Sq	EGFR	PR	490	0	Erlotinib	Osimertinib	Nivolumab				
10	75	F	non-Sq	EGFR	PD	168	1	Gefitinib	CDDP+PEM	Pembrolizumab				
11	50	F	non-Sq	EGFR	PR	159	0	Erlotinib	CDDP+PEM	Pembrolizumab				
12	55	M	non-Sq	EGFR	PR	218	0	CBDCA+PTX	Afatinib	Osimertinib	Pembrolizumab			

CBDCA: Carboplatin, CDDP: Cisplatin, PEM: Pemetrexed, VNR: Vinorelbine, PTX: Paclitaxel, DTX: Docetaxel, Bev: Bevacizumab

Supplementary Figure 2

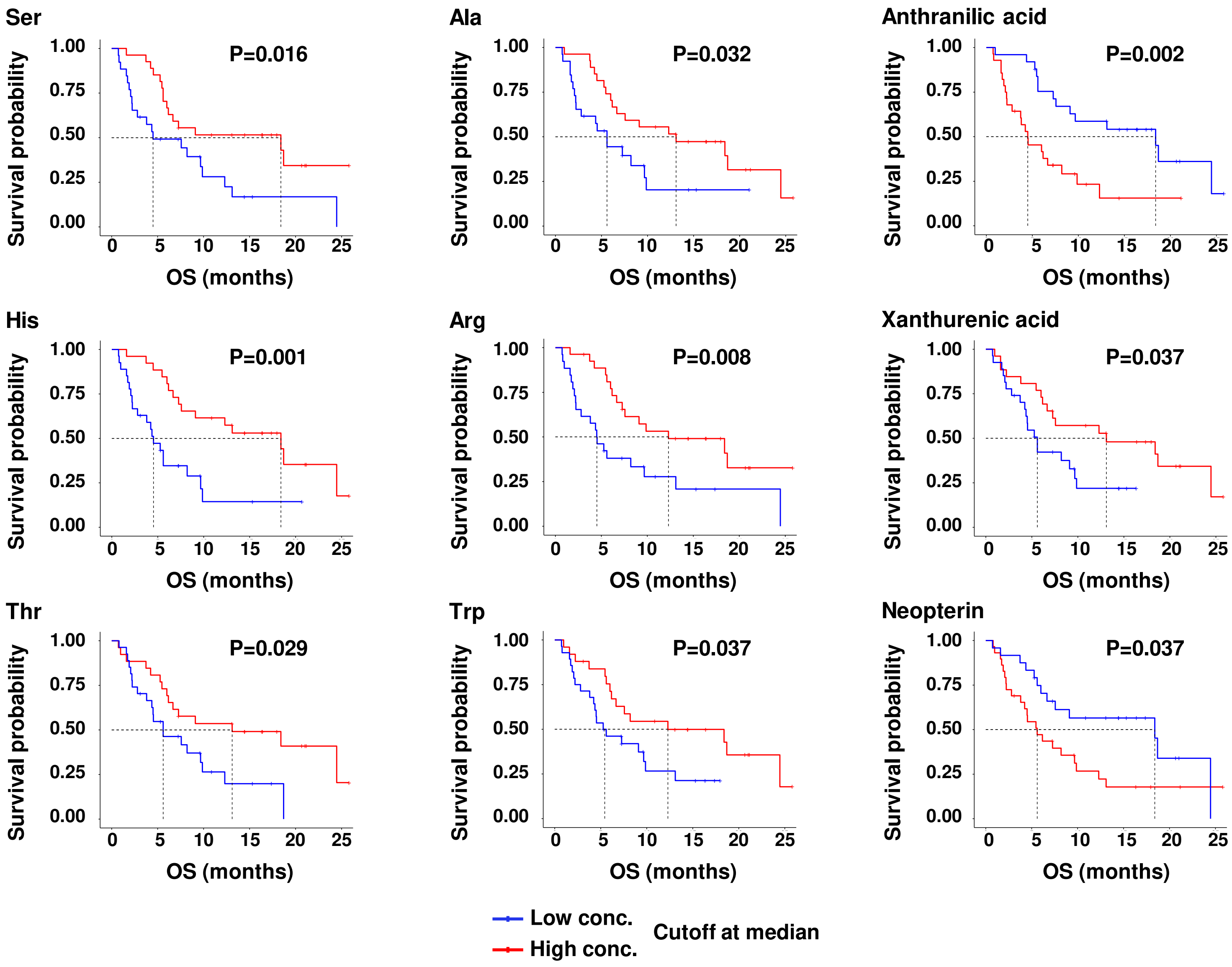

Supplementary Figure 3

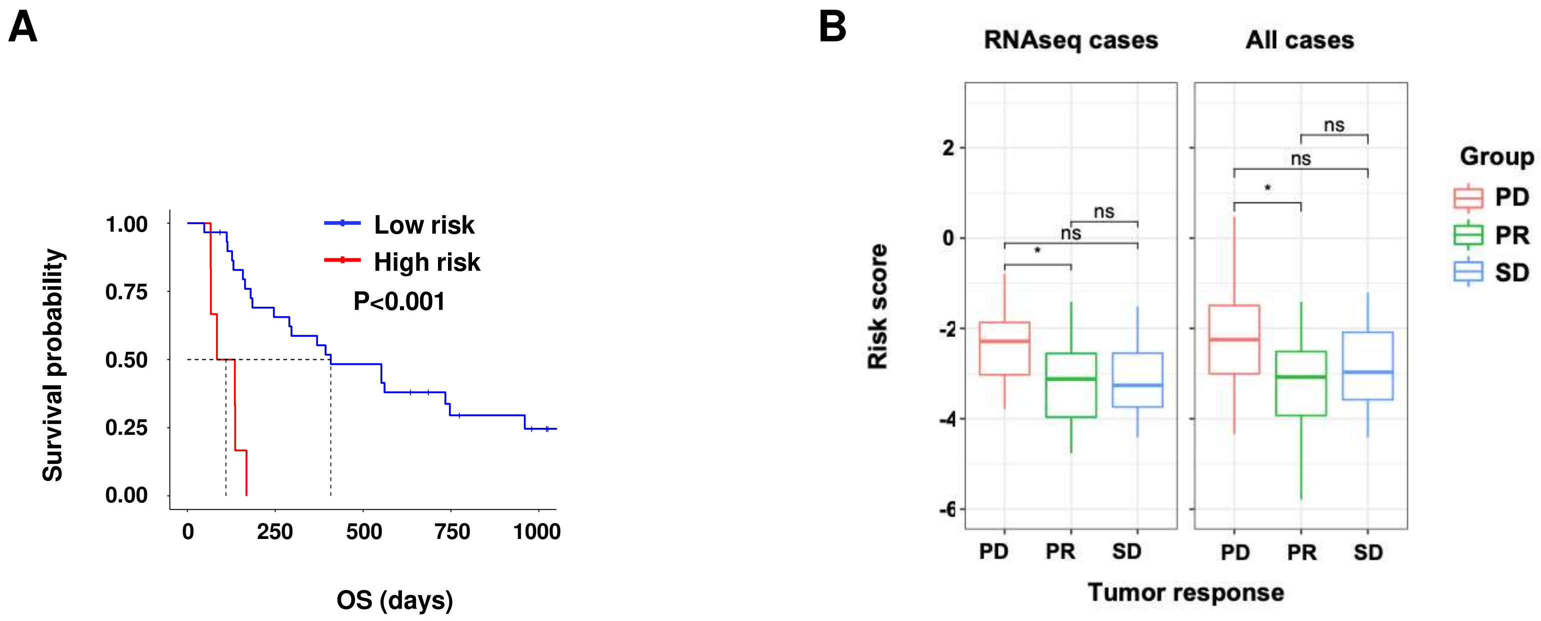

*P<0.05; Mann–Whitney–Wilcoxon test.

Supplementary Figure 4

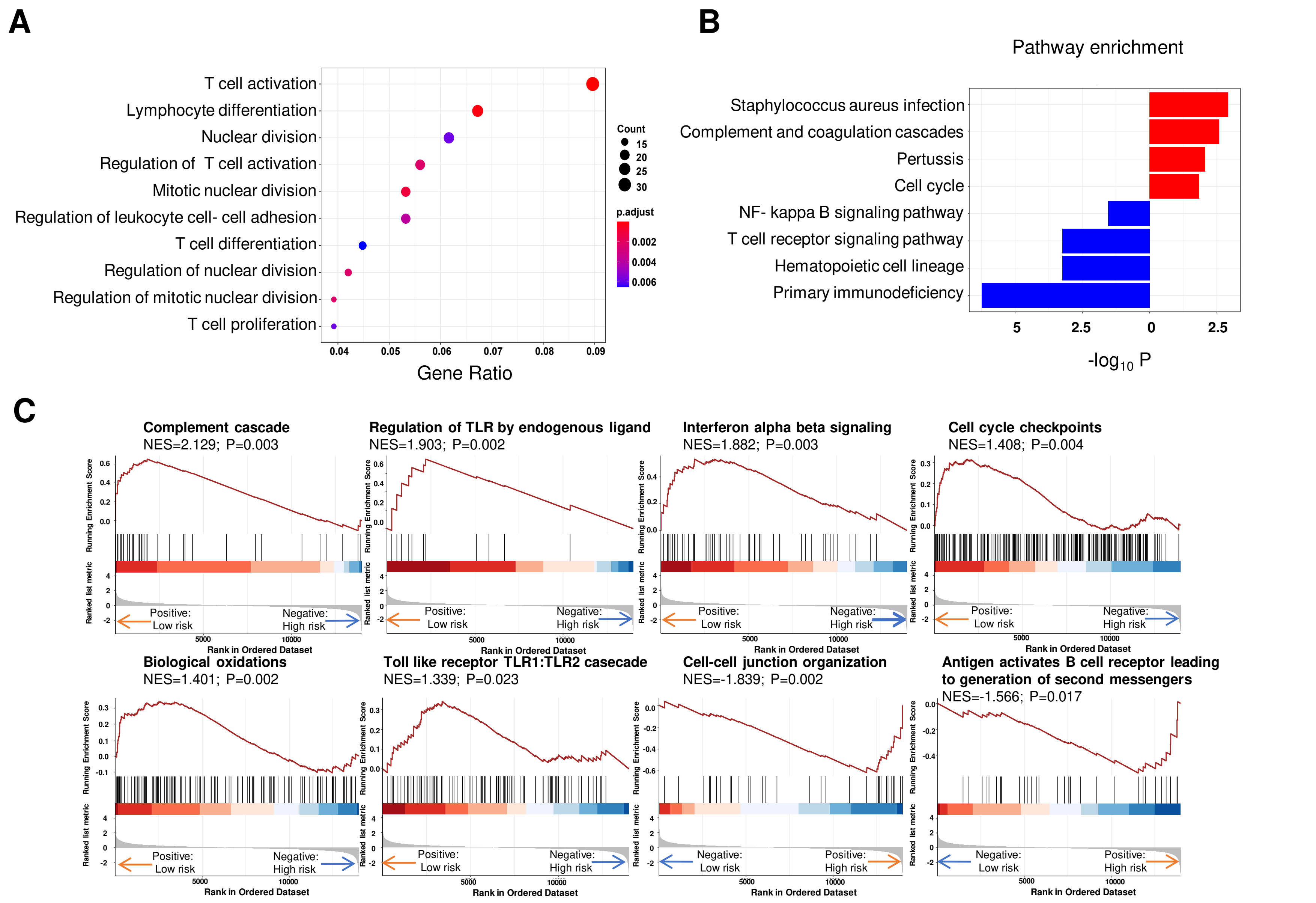

Supplementary Figure 5

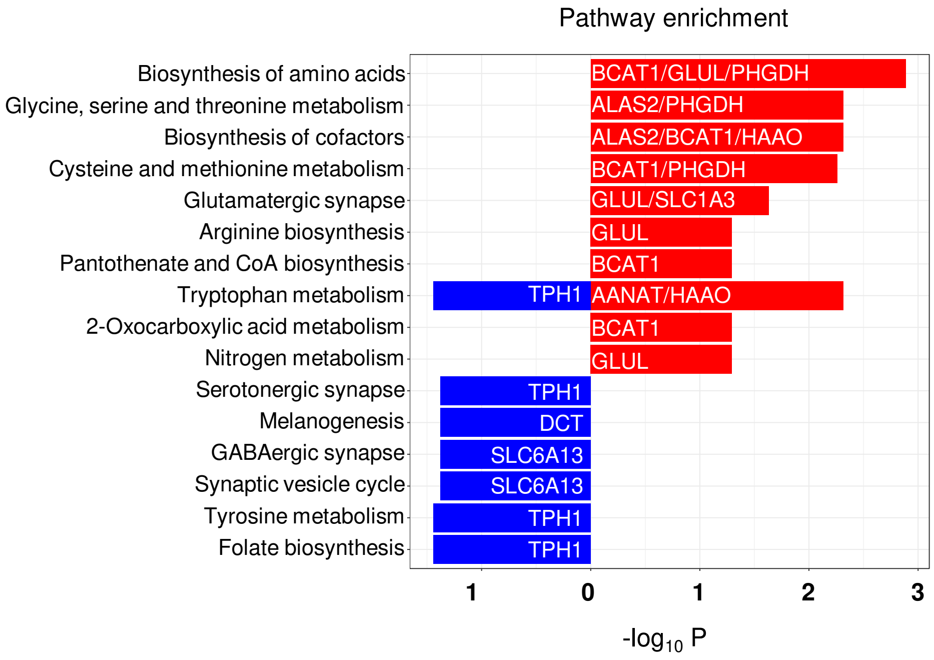

Supplementary Figure 6

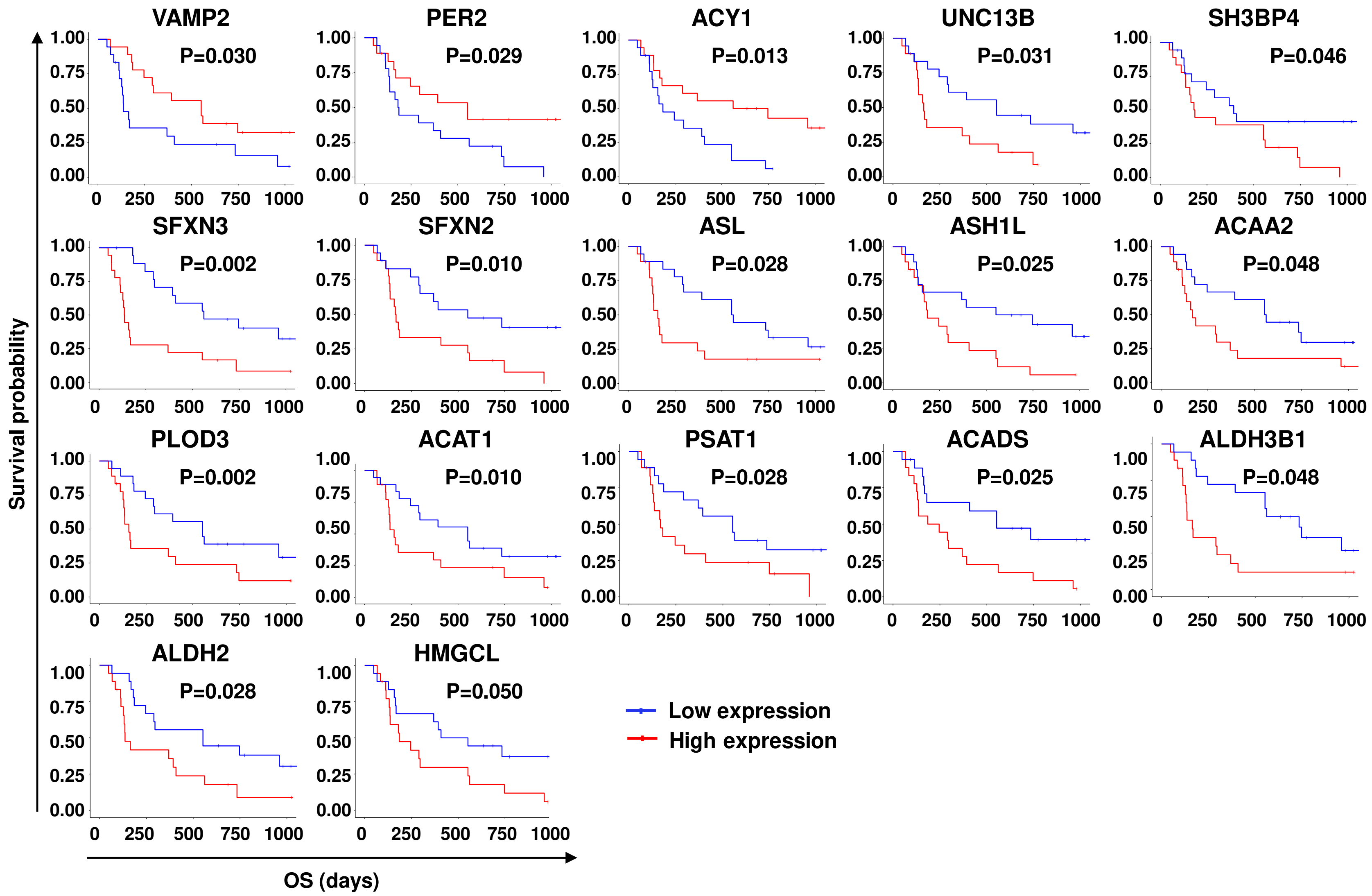

Supplementary Figure 7

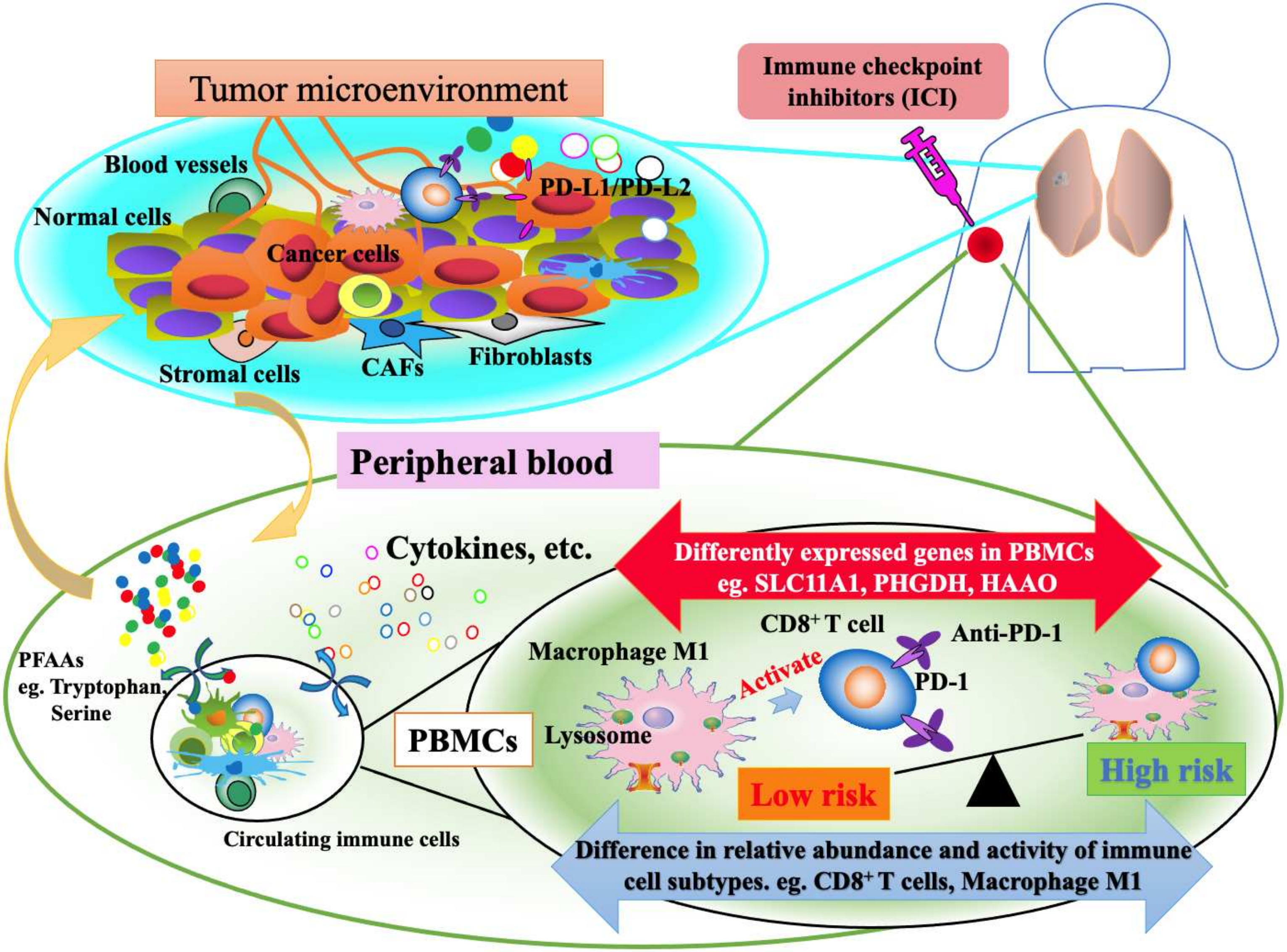

Supplement: Supplementary data [file jitc-2021-004420supp001.pdf]
