# Supplementary material for: Clinical significance of plasma-free amino acids and tryptophan metabolites in patients with non-small cell lung cancer receiving PD-1 inhibitor: a pilot cohort study for developing a prognostic multivariate model
Source: J Immunother Cancer. 2022 May 11;10(5):e004420. doi: 10.1136/jitc-2021-004420 (PMC9109096; doi:10.1136/jitc-2021-004420)
Supplement: Supplementary data [file jitc-2021-004420supp002.pdf]

## Supplementary Tables

### Supplementary Table 1. Characteristics of patients for RNA sequencing

| Patient characteristics | Total (N=36) |
|---|---|
| **Age (years), Mean (SD)** | 70.4 (7.1) |
| **Sex, N (%)** | |
| **Female** | 8 (22%) |
| **Male** | 28 (78%) |
| **Smoking, N (%)** | |
| **Former** | 27 (75%) |
| **Never** | 9 (25%) |
| **Tumor stage, N (%)** | |
| **Recurrent** | 4 (11%) |
| **Stage IV** | 32 (89%) |
| **Performance status, N (%)** | |
| **0-1** | 15 (42%) |
| **2-3** | 21 (58%) |
| **Histology, N (%)** | |
| **non-Squamous** | 25 (69%) |
| **Squamous** | 11 (31%) |
| **Driver mutation, N (%)** | |
| **WT** | 29 (81%) |
| **EGFR** | 6 (17%) |
| **ALK** | 1 (2.8%) |
| **Tumor PD-L1 expression, N (%)** | |
| **0~49 %** | 9 (29%) |
| **50~100 %** | 22 (71%) |
| **Treatment line, N (%)** | |
| **1st line** | 12 (33%) |
| **2~7 line** | 24 (67%) |
| **PD-1 blocker, N (%)** | |
| **Nivolumab** | 20 (56%) |
| **Pembrolizumab** | 16 (44%) |
| **Blood test, Mean (SD)** | |

| | |
|---|---|
| **Albumin (g/dL)** | 3.40 (0.53) |
| **LDH (U/L)** | 255 (93.8) |
| **WBC (/μL)** | 7592 (3600) |
| **Lymphocyte (/μL)** | 1438 (677) |
| **Neutrophil (/μL)** | 5481 (3199) |
| **Eosinophil (/μL)** | 173 (180) |
| **Monocytes (/μL)** | 462 (212) |
| **NLR** | 4.82 (3.67) |

The clinical characteristics of patients for RNA sequencing were described. Continuous variables are shown as mean and standard deviation. Categorical variables are shown as sample numbers and percentages.

**Supplementary Table 2. PFAA and metabolite concentrations before ICI therapy in patients for RNA sequencing**

| PFAA/metabolite (μM) | Mean (SD) |
|---|---|
| **Glutamic Acid** | 51.8 (23.0) |
| **Serine** | 101 (25.7) |
| **Asparagine** | 40.3 (7.7) |
| **Glycine** | 213.0 (66.7) |
| **Glutamine** | 51.8 (23.0) |
| **Histidine** | 65.7 (24.3) |
| **Threonine** | 106.0 (30.6) |
| **Alanine** | 302.0 (98.3) |
| **Citrulline** | 29.9 (10.7) |
| **Arginine** | 74.3 (18.0) |
| **Proline** | 146.0 (54.3) |
| **α-Amino Butyric Acid** | 16.6 (4.9) |
| **Tyrosine** | 62.7 (14.1) |
| **Valine** | 208.0 (50.7) |
| **Methionine** | 21.1 (4.7) |
| **Ornithine** | 65.0 (11.7) |
| **Lysine** | 173.0 (45.7) |
| **Isoleucine** | 65.7 (15.6) |
| **Leucine** | 117.0 (31.2) |

| | |
|---|---|
| **Phenylalanine** | 60.4 (12.2) |
| **Tryptophan** | 45.9 (11.9) |
| **3h-Kynurenine** | 0.06 (0.03) |
| **3h-Anthranilic Acid** | 0.04 (0.02) |
| **5h-Indol-3-Acetic Acid** | 0.06 (0.03) |
| **5h-Tryptophan** | 0.00 (0.00) |
| **Anthranilic Acid** | 0.02 (0.02) |
| **Indol-3-Acetic Acid** | 2.06 (2.07) |
| **Indol-3-Lactic Acid** | 0.81 (0.50) |
| **Kynurenic Acid** | 0.05 (0.02) |
| **Kynurenine** | 2.34 (0.68) |
| **Picolinic Acid** | 0.05 (0.03) |
| **Quinolinic Acid** | 0.67 (0.43) |
| **Serotonin** | 0.14 (0.15) |
| **Xanthurenic Acid** | 0.01 (0.01) |
| **Neopterine** | 0.01 (0.01) |
| **N'-Formyl-Kynurenine** | 0.02 (0.02) |

Mean and standard deviation are shown.

## Supplementary Table 3. Go enrichment of genes differentially expressed between the high- and low-risk groups

| ID | Description | P | P.adj | gene ID |
|---|---|---|---|---|
| **GO:0042110** | **T cell activation** | 0.000 | 0.000 | AIF1/CD151/CEBPB/IL10/LGALS9/LILRB2/SFTPD/SLC11A1/SOX4/VSIG4/ZBTB16/BCL11B/BTLA/CD2/CD3D/CD3G/CD40LG/CD5/CD6/CD8A/CD8B/HES1/HHLA2/HSPH1/IL20RB/IL7R/LCK/LY9/RASGRP1/RHOH/RORA/THEMIS |
| **GO:0030098** | **lymphocyte differentiation** | 0.000 | 0.000 | IL10/LGALS9/LILRB2/PGLYRP1/SOX4/SPI1/ZBTB16/BCL11B/BLNK/CD2/CD3D/CD3G/CD40LG/CD8A/CR2/IKZF3/IL7R/LCK/LY9/PCID2/RASGRP1/RHOH/RORA/THEMIS |
| **GO:0140014** | **mitotic nuclear division** | 0.000 | 0.001 | AURKB/BIRC5/CDC6/CDCA5/CDT1/EPGN/HSPA1B/INSR/KIF18B/KIF4A/MKI67/NUSAP1/PKMYT1/PLK1/PSRC1/TPX2/UBE2C/MAP9/PCID2 |
| **GO:0050863** | **regulation of T cell activation** | 0.000 | 0.002 | AIF1/CEBPB/IL10/LGALS9/LILRB2/SFTPD/VSIG4/ZBTB16/BTLA/CD2/CD40LG/CD5/CD6/HES1/HHLA2/HSPH1/IL20RB/IL7R/LCK/RASGRP1 |
| **GO:0007088** | **regulation of mitotic nuclear division** | 0.000 | 0.002 | AURKB/CDC6/CDCA5/CDT1/EPGN/HSPA1B/INSR/MKI67/NUSAP1/PKMYT1/PLK1/UBE2C/MAP9/PCID2 |
| **GO:0051783** | **regulation of nuclear division** | 0.000 | 0.002 | AURKB/CDC6/CDCA5/CDT1/EPGN/FBXO43/HSPA1B/INSR/MKI67/NUSAP1/PKMYT1/PLK1/UBE2C/MAP9/PCID2 |
| **GO:1903037** | **regulation of leukocyte cell-cell adhesion** | 0.000 | 0.004 | AIF1/CEBPB/IL10/LGALS9/LILRB2/SFTPD/VSIG4/ZBTB16/BTLA/CD40LG/CD5/CD6/HES1/HHLA2/HSPH1/IL20RB/IL7R/LCK/RASGRP1 |
| **GO:0042098** | **T cell proliferation** | 0.000 | 0.006 | AIF1/CD151/CEBPB/IL10/LGALS9/LILRB2/SFTPD/SLC11A1/VSIG4/CD40LG/CD6/HES1/HHLA2/IL20RB |
| **GO:0000280** | **nuclear division** | 0.000 | 0.006 | AURKB/BIRC5/CDC6/CDCA5/CDT1/EPGN/FBXO43/HSPA1B/INSR/KIF18B/KIF4A/MKI67/MND1/NUSAP1/PKMYT1/PLK1/PSRC1/TOP2A/TPX2/UBE2C/MAP9/PCID2 |
| **GO:0030217** | **T cell differentiation** | 0.000 | 0.007 | LGALS9/LILRB2/SOX4/ZBTB16/BCL11B/CD2/CD3D/CD3G/CD8A/IL7R/LCK/LY9/RASGRP1/RHOH/RORA/THEMIS |
| **GO:0097421** | **liver regeneration** | 0.000 | 0.007 | CEBPB/IL10/TYMS/CCND1/PTCH1/WDR35 |

| | | | | |
|---|---|---|---|---|
| **GO:0046651** | **lymphocyte proliferation** | 0.000 | 0.007 | AIF1/CD151/CEBPB/IL10/LGALS9/LILRB2/SFTPD/SLC11A1/VSIG4/CD40LG/CD6/CR2/HES1/HHLA2/IKZF3/IL20RB/IL7R |
| **GO:0032943** | **mononuclear cell proliferation** | 0.000 | 0.007 | AIF1/CD151/CEBPB/IL10/LGALS9/LILRB2/SFTPD/SLC11A1/VSIG4/CD40LG/CD6/CR2/HES1/HHLA2/IKZF3/IL20RB/IL7R |
| **GO:0001754** | **eye photoreceptor cell differentiation** | 0.000 | 0.007 | CABP4/MYO7A/ROM1/CCDC66/CEP290/PRKCI/RORB |
| **GO:0007159** | **leukocyte cell-cell adhesion** | 0.000 | 0.008 | AIF1/CEBPB/IL10/LGALS9/LILRB2/SFTPD/VSIG4/ZBTB16/BTLA/CD40LG/CD5/CD6/HES1/HHLA2/HSPH1/IL20RB/IL7R/LCK/RASGRP1 |
| **GO:0007052** | **mitotic spindle organization** | 0.000 | 0.008 | AURKB/BIRC5/HSPA1B/KIF4A/PLK1/PSRC1/SPC25/STMN1/TPX2/MAP9 |
| **GO:0050870** | **positive regulation of T cell activation** | 0.000 | 0.008 | AIF1/LGALS9/LILRB2/ZBTB16/BTLA/CD40LG/CD5/CD6/HES1/HHLA2/HSPH1/IL7R/LCK/RASGRP1 |
| **GO:0030278** | **regulation of ossification** | 0.000 | 0.008 | CEBPB/JAG1/LTF/OMD/ZBTB16/ADRB2/AXIN2/ID3/NOG/PTCH1/RORB/S1PR1/TOB2/TPH1 |
| **GO:0003007** | **heart morphogenesis** | 0.000 | 0.009 | CPE/FOXC1/INSR/JAG1/MYBPC3/PKP2/SOX4/AXIN2/BBS7/CCDC40/EFNA1/HES1/NOG/PTCH1/S1PR1/TNNC1 |
| **GO:1902850** | **microtubule cytoskeleton organization involved in mitosis** | 0.000 | 0.009 | AURKB/BIRC5/HSPA1B/KIF4A/NUSAP1/PLK1/PSRC1/SPC25/STMN1/TPX2/MAP9 |
| **GO:0042129** | **regulation of T cell proliferation** | 0.000 | 0.009 | AIF1/CEBPB/IL10/LGALS9/LILRB2/SFTPD/VSIG4/CD40LG/CD6/HES1/HHLA2/IL20RB |
| **GO:0042462** | **eye photoreceptor cell development** | 0.000 | 0.009 | CABP4/MYO7A/CCDC66/CEP290/PRKCI/RORB |
| **GO:0048285** | **organelle fission** | 0.000 | 0.010 | AURKB/BIRC5/CDC6/CDCA5/CDT1/EPGN/FBXO43/HSPA1B/INSR/KIF18B/KIF4A/MKI67/MND1/NUSAP1/PKMYT1/PLK1/PSRC1/TOP2A/TPX2/UBE2C/MAP9/PCID2 |
| **GO:0051249** | **regulation of lymphocyte activation** | 0.000 | 0.010 | AIF1/CEBPB/IL10/LGALS9/LILRB2/PGLYRP1/SFTPD/VSIG4/ZBTB16/BTLA/CD2/CD40LG/CD5/CD6/HES1/HHLA2/HSPH1/IKZF3/IL20RB/IL7R/LCK/PCID2/RASGRP1 |
| **GO:0070661** | **leukocyte proliferation** | 0.000 | 0.010 | AIF1/CD151/CEBPB/IL10/LGALS9/LILRB2/SFTPD/SLC11A1/VSIG4/CD40LG/CD6/CR2/HES1/HHLA2/IKZF3/IL20RB/IL7R |
| **GO:0045931** | **positive regulation of mitotic cell cycle** | 0.000 | 0.010 | AIF1/CDC6/CDCA5/CDK1/CDT1/EPGN/INSR/NUSAP1/UBE2C/CCND1/HES1/PCID2 |
| **GO:1902105** | **regulation of leukocyte differentiation** | 0.000 | 0.010 | C1QC/CEBPB/LGALS9/LILRB2/LTF/NME1/PGLYRP1/PPARGC1B/ZBTB16/CD2/IKZF3/IL7R/PCID2/RASGRP1/ROR2/TOB2 |
| **GO:0003208** | **cardiac ventricle morphogenesis** | 0.000 | 0.010 | CPE/FOXC1/JAG1/MYBPC3/PKP2/SOX4/NOG/TNNC1 |
| **GO:0031100** | **animal organ regeneration** | 0.000 | 0.010 | ADM/CDK1/CEBPB/IL10/TYMS/CCND1/PTCH1/WDR35 |
| **GO:0007093** | **mitotic cell cycle checkpoint** | 0.000 | 0.011 | AURKB/CDC6/CDK1/CDT1/CLSPN/E2F1/PLK1/SOX4/TOP2A/BRSK1/CCND1/PCID2 |
| **GO:1903039** | **positive regulation of leukocyte cell-cell adhesion** | 0.000 | 0.011 | AIF1/LGALS9/LILRB2/ZBTB16/BTLA/CD40LG/CD5/CD6/HES1/HHLA2/HSPH1/IL7R/LCK/RASGRP1 |
| **GO:0090307** | **mitotic spindle assembly** | 0.000 | 0.011 | AURKB/BIRC5/HSPA1B/KIF4A/PLK1/TPX2/MAP9 |
| **GO:0022407** | **regulation of cell-cell adhesion** | 0.000 | 0.012 | AIF1/CEBPB/IL10/JAG1/LGALS9/LILRB2/SFTPD/VSIG4/ZBTB16/BTLA/CD40LG/CD5/CD6/HES1/HHLA2/HSPH1/IL20RB/IL7R/LCK/RASGRP1 |
| **GO:0051098** | **regulation of binding** | 0.000 | 0.013 | APOE/AURKB/CDCA5/CDT1/E2F1/FOXC1/HJURP/IL10/NME1/P2RY1/PLK1/SLPI/STMN1/ADRB2/ENHO/HABP4/HES1/ID3/NOG |
| **GO:0051384** | **response to glucocorticoid** | 0.000 | 0.013 | AANAT/ADM/AIF1/ALPL/DDIT4/IL10/PPARGC1B/TYMS/ABCC2/CCND1/UCP3 |
| **GO:0022409** | **positive regulation of cell-cell adhesion** | 0.000 | 0.014 | AIF1/IL10/LGALS9/LILRB2/ZBTB16/BTLA/CD40LG/CD5/CD6/HES1/HHLA2/HSPH1/IL7R/LCK/RASGRP1 |
| **GO:0042130** | **negative regulation of T cell proliferation** | 0.000 | 0.014 | CEBPB/IL10/LGALS9/LILRB2/SFTPD/VSIG4/IL20RB |
| **GO:0051101** | **regulation of DNA binding** | 0.000 | 0.014 | CDCA5/CDT1/E2F1/FOXC1/HJURP/NME1/ENHO/HABP4/HES1/ID3 |
| **GO:0002449** | **lymphocyte mediated immunity** | 0.000 | 0.016 | C1QA/C1QB/C1QC/C2/EXO1/IL10/JAG1/KIR3DL1/LGALS9/SERPING1/SLC11A1/CD40LG/CD8A/CR2/IL20RB/IL7R/LTA/RASGRP1 |
| **GO:0007059** | **chromosome segregation** | 0.000 | 0.016 | AURKB/BIRC5/CDC6/CDCA5/CDT1/HJURP/KIF18B/KIF4A/MKI67/NUSAP1/PLK1/PSRC1/SKA3/SPC25/TOP2A/AXIN2/PCID2 |
| **GO:0006959** | **humoral immune response** | 0.000 | 0.017 | C1QA/C1QB/C1QC/C2/CAMP/DEFA3/EXO1/LTF/PGLYRP1/SERPING1/SFTPD/SLC11A1/SLPI/VSIG4/BLNK/CR2/LTA/PRSS3 |
| **GO:0045787** | **positive regulation of cell cycle** | 0.000 | 0.017 | AIF1/AURKB/CDC6/CDCA5/CDK1/CDT1/E2F1/EPGN/HMGN5/IL10/INSR/NUSAP1/PSRC1/SOX4/UBE2C/CCND1/CXCR5/HES1/PCID2 |
| **GO:0050670** | **regulation of lymphocyte proliferation** | 0.000 | 0.017 | AIF1/CEBPB/IL10/LGALS9/LILRB2/SFTPD/VSIG4/CD40LG/CD6/HES1/HHLA2/IKZF3/IL20RB |
| **GO:0046530** | **photoreceptor cell differentiation** | 0.000 | 0.017 | CABP4/MYO7A/ROM1/CCDC66/CEP290/PRKCI/RORB |
| **GO:0042136** | **neurotransmitter biosynthetic process** | 0.000 | 0.017 | AIF1/GCHFR/IL10/INSR/SLC1A3/RORA/SLC44A4/TPH1/WDR35 |
| **GO:0003231** | **cardiac ventricle development** | 0.000 | 0.017 | CPE/FOXC1/JAG1/MYBPC3/PKP2/SOX4/HES1/NOG/SALL4/TNNC1 |
| **GO:0032944** | **regulation of mononuclear cell proliferation** | 0.000 | 0.017 | AIF1/CEBPB/IL10/LGALS9/LILRB2/SFTPD/VSIG4/CD40LG/CD6/HES1/HHLA2/IKZF3/IL20RB |
| **GO:0000083** | **regulation of transcription involved in G1/S transition of mitotic cell cycle** | 0.000 | 0.017 | CDC6/CDT1/E2F1/TYMS/GFI1 |
| **GO:0031348** | **negative regulation of defense response** | 0.000 | 0.017 | APOE/GPX1/IL10/IL1R2/LGALS9/NR1H3/PGLYRP1/SERPING1/VSIG4/WFDC1/ABCD2/CD200/IL20RB/RORA |
| **GO:0032496** | **response to lipopolysaccharide** | 0.000 | 0.017 | ADM/ALPL/CEBPB/DEFA3/IL10/LGALS9/LILRB2/LTF/NR1H3/SLC11A1/SLPI/TIMP4/ABCC2/CD6/GFI1/LTA/WDR35 |
| **GO:0031570** | **DNA integrity checkpoint** | 0.000 | 0.017 | CDC6/CDK1/CDT1/CLSPN/E2F1/PLK1/SOX4/TOP2A/BRSK1/CCND1 |

| | | | | |
|---|---|---|---|---|
| | | | | /USP28 |
| **GO:0002437** | **inflammatory response to antigenic stimulus** | 0.000 | 0.018 | GPX1/IL10/CD6/IL20RB/LTA/RASGRP1 |
| **GO:0042461** | **photoreceptor cell development** | 0.000 | 0.019 | CABP4/MYO7A/CCDC66/CEP290/PRKCI/RORB |
| **GO:0032102** | **negative regulation of response to external stimulus** | 0.000 | 0.020 | AIF1/APOE/GPX1/IL10/IL1R2/LGALS9/LTF/NR1H3/PGLYRP1/SERPING1/VSIG4/WFDC1/ABCD2/CACNB3/CD200/IL20RB/PLAT/RORA/SEMA3A |
| **GO:0000075** | **cell cycle checkpoint** | 0.000 | 0.020 | AURKB/CDC6/CDK1/CDT1/CLSPN/E2F1/PLK1/SOX4/TOP2A/BRSK1/CCND1/PCID2/USP28 |
| **GO:0002683** | **negative regulation of immune system process** | 0.000 | 0.020 | C1QC/CEBPB/GPX1/IL10/LGALS9/LILRB2/LTF/NME1/NR1H3/PGLYRP1/SERPING1/SFTPD/VSIG4/ZBTB16/CD200/HES1/IL20RB/IL7R/TOB2/UBASH3A |
| **GO:0000819** | **sister chromatid segregation** | 0.000 | 0.020 | AURKB/CDC6/CDCA5/CDT1/KIF18B/KIF4A/NUSAP1/PLK1/PSRC1/TOP2A/AXIN2/PCID2 |
| **GO:0031960** | **response to corticosteroid** | 0.000 | 0.020 | AANAT/ADM/AIF1/ALPL/DDIT4/IL10/PPARGC1B/TYMS/ABCC2/CCND1/UCP3 |
| **GO:1903706** | **regulation of hemopoiesis** | 0.000 | 0.023 | C1QC/CEBPB/FOXC1/HSPA1B/JAG1/LGALS9/LILRB2/LTF/NME1/PGLYRP1/PPARGC1B/SPI1/ZBTB16/CD2/HES1/IKZF3/IL7R/PCID2/RASGRP1/ROR2/TOB2 |
| **GO:0002237** | **response to molecule of bacterial origin** | 0.000 | 0.023 | ADM/ALPL/CEBPB/DEFA3/IL10/LGALS9/LILRB2/LTF/NR1H3/SLC11A1/SLPI/TIMP4/ABCC2/CD6/GFI1/LTA/WDR35 |
| **GO:0070663** | **regulation of leukocyte proliferation** | 0.000 | 0.024 | AIF1/CEBPB/IL10/LGALS9/LILRB2/SFTPD/VSIG4/CD40LG/CD6/HES1/HHLA2/IKZF3/IL20RB |
| **GO:0050678** | **regulation of epithelial cell proliferation** | 0.000 | 0.024 | APOE/ATOH8/EPGN/GLUL/GPX1/IL10/NME1/WFDC1/BCL11B/CCND1/CDH13/HES1/IQGAP3/NOG/PLCG1/PTCH1/TACSTD2/WNT7A |
| **GO:0072593** | **reactive oxygen species metabolic process** | 0.000 | 0.025 | AIF1/DDIT4/GCHFR/GPX1/HBE1/HBG1/HP/IFI6/IL10/INSR/NQO2/SFTPD/ABCD2/RORA/WDR35 |
| **GO:0050806** | **positive regulation of synaptic transmission** | 0.000 | 0.025 | APOE/GLUL/GRIN2C/LILRB2/SLC1A3/ADRB2/CACNB3/MPP2/NLGN2/ROR2/WNT7A |
| **GO:0050728** | **negative regulation of inflammatory response** | 0.000 | 0.025 | APOE/GPX1/IL10/IL1R2/NR1H3/PGLYRP1/WFDC1/ABCD2/CD200/IL20RB/RORA |
| **GO:0051100** | **negative regulation of binding** | 0.000 | 0.025 | AURKB/E2F1/IL10/P2RY1/SLPI/STMN1/ADRB2/ENHO/HABP4/ID3/NOG |
| **GO:0043368** | **positive T cell selection** | 0.000 | 0.025 | BCL11B/CD3D/CD3G/LY9/THEMIS |
| **GO:0048545** | **response to steroid hormone** | 0.000 | 0.025 | AANAT/ADM/AIF1/ALPL/DDIT4/DEFA3/IL10/NR1H3/PPARGC1B/TYMS/ABCC2/CCND1/GPAM/LBH/PMEPA1/RORA/RORB/UCP3 |
| **GO:0000281** | **mitotic cytokinesis** | 0.000 | 0.025 | CDT1/KIF4A/NUSAP1/PLK1/RHOC/STMN1/MAP9 |
| **GO:0035019** | **somatic stem cell population maintenance** | 0.000 | 0.025 | SOX4/SPI1/HES1/LBH/NOG/SALL4/WNT7A |
| **GO:0042116** | **macrophage activation** | 0.000 | 0.026 | AIF1/C1QA/IL10/NR1H3/SLC11A1/VSIG4/CD200/RORA |
| **GO:0032945** | **negative regulation of mononuclear cell proliferation** | 0.000 | 0.026 | CEBPB/IL10/LGALS9/LILRB2/SFTPD/VSIG4/IL20RB |
| **GO:0050672** | **negative regulation of lymphocyte proliferation** | 0.000 | 0.026 | CEBPB/IL10/LGALS9/LILRB2/SFTPD/VSIG4/IL20RB |
| **GO:0045840** | **positive regulation of mitotic nuclear division** | 0.001 | 0.026 | CDCA5/CDT1/EPGN/INSR/NUSAP1/UBE2C |
| **GO:0060563** | **neuroepithelial cell differentiation** | 0.001 | 0.026 | CEBPB/JAG1/MYO7A/SOX4/HES1/SLC4A7 |
| **GO:0070228** | **regulation of lymphocyte apoptotic process** | 0.001 | 0.026 | AURKB/IL10/LGALS9/CD3G/EFNA1/IL7R |
| **GO:0050867** | **positive regulation of cell activation** | 0.001 | 0.032 | AIF1/IL10/LGALS9/LILRA5/LILRB2/ZBTB16/BTLA/CD2/CD40LG/CD5/CD6/HES1/HHLA2/HSPH1/IL7R/LCK/PCID2/RASGRP1 |
| **GO:0002460** | **adaptive immune response based on somatic recombination of immune receptors built from immunoglobulin superfamily domains** | 0.001 | 0.032 | C1QA/C1QB/C1QC/C2/EXO1/IL10/JAG1/SERPING1/SLC11A1/CD40LG/CD8A/CR2/IL20RB/IL7R/LTA/LY9/RORA |
| **GO:0060236** | **regulation of mitotic spindle organization** | 0.001 | 0.032 | HSPA1B/PLK1/PSRC1/TPX2/MAP9 |
| **GO:0090068** | **positive regulation of cell cycle process** | 0.001 | 0.032 | AIF1/AURKB/CDC6/CDCA5/CDK1/CDT1/E2F1/EPGN/INSR/NUSAP1/SOX4/UBE2C/CCND1/CXCR5/PCID2 |
| **GO:0001819** | **positive regulation of cytokine production** | 0.001 | 0.032 | AGPAT2/AIF1/CEBPB/HSPA1B/IL10/LGALS9/LILRA5/LILRB2/SLC11A1/CD2/CD200/CD40LG/CD6/HHLA2/IL20RB/LTA/LY9/MCOLN2/RASGRP1/RORA |
| **GO:0000070** | **mitotic sister chromatid segregation** | 0.001 | 0.032 | AURKB/CDC6/CDCA5/CDT1/KIF18B/KIF4A/NUSAP1/PLK1/PSRC1/PCID2 |
| **GO:0061640** | **cytoskeleton-dependent cytokinesis** | 0.001 | 0.032 | AURKB/CDT1/KIF4A/NUSAP1/PLK1/RHOC/STMN1/MAP9 |
| **GO:0001503** | **ossification** | 0.001 | 0.033 | ALPL/CEBPB/FOXC1/JAG1/LTF/OMD/PPARGC1B/ZBTB16/ADRB2/AXIN2/ID3/NOG/PTCH1/ROR2/RORB/S1PR1/TOB2/TPH1 |
| **GO:0032649** | **regulation of interferon-gamma production** | 0.001 | 0.033 | IL10/LGALS9/PGLYRP1/SLC11A1/CD2/IL20RB/LTA/RASGRP1 |
| **GO:0070664** | **negative regulation of leukocyte proliferation** | 0.001 | 0.033 | CEBPB/IL10/LGALS9/LILRB2/SFTPD/VSIG4/IL20RB |
| **GO:0042133** | **neurotransmitter metabolic process** | 0.001 | 0.033 | AIF1/GCHFR/IL10/INSR/PHGDH/SLC1A3/RORA/SLC44A4/TPH1/WDR35 |

| | | | | |
|---|---|---|---|---|
| **GO:0048732** | **gland development** | 0.001 | 0.033 | CCNB2/CEBPB/FOXC1/GPX1/IL10/INSR/NME1/TYMS/BCL11B/CCDC40/CCND1/HES1/IQGAP3/LBH/NOG/PTCH1/SEMA3A/TPH1/WDR35 |
| **GO:0050673** | **epithelial cell proliferation** | 0.001 | 0.033 | APOE/ATOH8/CEBPB/EPGN/GLUL/GPX1/IL10/NME1/WFDC1/BCL11B/CCND1/CDH13/HES1/IQGAP3/NOG/PLCG1/PTCH1/TACSTD2/WNT7A |
| **GO:0051983** | **regulation of chromosome segregation** | 0.001 | 0.036 | AURKB/CDC6/CDCA5/CDT1/MKI67/PLK1/AXIN2/PCID2 |
| **GO:0033045** | **regulation of sister chromatid segregation** | 0.001 | 0.036 | AURKB/CDC6/CDCA5/CDT1/PLK1/AXIN2/PCID2 |
| **GO:0003206** | **cardiac chamber morphogenesis** | 0.001 | 0.036 | CPE/FOXC1/JAG1/MYBPC3/PKP2/SOX4/HES1/NOG/TNNC1 |
| **GO:0032753** | **positive regulation of interleukin-4 production** | 0.001 | 0.037 | CEBPB/LGALS9/CD40LG/IL20RB |
| **GO:0051043** | **regulation of membrane protein ectodomain proteolysis** | 0.001 | 0.037 | APOE/IL10/TIMP2/TIMP4 |
| **GO:1903708** | **positive regulation of hemopoiesis** | 0.001 | 0.037 | FOXC1/HSPA1B/JAG1/LGALS9/LILRB2/PPARGC1B/ZBTB16/IL7R/PCID2/RASGRP1/ROR2 |
| **GO:0035315** | **hair cell differentiation** | 0.001 | 0.039 | JAG1/MYO7A/HES1/SLC44A4/SLC4A7 |
| **GO:0044774** | **mitotic DNA integrity checkpoint** | 0.001 | 0.040 | CDC6/CDK1/CLSPN/E2F1/SOX4/TOP2A/BRSK1/CCND1 |
| **GO:0098883** | **synapse pruning** | 0.001 | 0.041 | C1QA/C1QB/C1QC |
| **GO:0002861** | **regulation of inflammatory response to antigenic stimulus** | 0.001 | 0.041 | GPX1/IL10/IL20RB/LTA |
| **GO:0090224** | **regulation of spindle organization** | 0.001 | 0.042 | HSPA1B/PLK1/PSRC1/TPX2/MAP9 |
| **GO:0034113** | **heterotypic cell-cell adhesion** | 0.001 | 0.042 | IL10/LILRB2/PKP2/CD2/CD200/LCK |
| **GO:0002696** | **positive regulation of leukocyte activation** | 0.001 | 0.043 | AIF1/IL10/LGALS9/LILRB2/ZBTB16/BTLA/CD2/CD40LG/CD5/CD6/HES1/HHLA2/HSPH1/IL7R/LCK/PCID2/RASGRP1 |
| **GO:0060219** | **camera-type eye photoreceptor cell differentiation** | 0.001 | 0.047 | CABP4/ROM1/CCDC66/RORB |
| **GO:0045444** | **fat cell differentiation** | 0.001 | 0.048 | CEBPB/E2F1/GPX1/JAG1/JDP2/ZBTB16/ADRB2/BBS7/CCND1/HES1/RORA/TPH1 |
| **GO:0002862** | **negative regulation of inflammatory response to antigenic stimulus** | 0.001 | 0.050 | GPX1/IL10/IL20RB |
| **GO:0007077** | **mitotic nuclear envelope disassembly** | 0.001 | 0.050 | CCNB2/CDK1/PLK1 |
| **GO:2001198** | **regulation of dendritic cell differentiation** | 0.001 | 0.050 | CEBPB/LGALS9/LILRB2 |

Go term enrichment results (P.adjust: BH method).

## Supplementary Table 4. KEGG pathway enrichment of genes differentially expressed between the high- and low-risk groups

| ID | Description | P | P.adj | geneID | group |
|---|---|---|---|---|---|
| **hsa05150** | **Staphylococcus aureus infection** | 0.000 | 0.001 | C1QA/C1QB/C1QC/C2/CAMP/DEFA3/FCGR3B/IL10 | 1 |
| **hsa04610** | **Complement and coagulation cascades** | 0.000 | 0.003 | C1QA/C1QB/C1QC/C2/SERPINA1/SERPING1/VSIG4 | 1 |
| **hsa05133** | **Pertussis** | 0.000 | 0.008 | C1QA/C1QB/C1QC/C2/IL10/SERPING1 | 1 |
| **hsa04110** | **Cell cycle** | 0.000 | 0.014 | CCNB2/CDC6/CDK1/E2F1/E2F2/PKMYT1/PLK1 | 1 |
| **hsa05340** | **Primary immunodeficiency** | 0.000 | 0.000 | BLNK/CD3D/CD40LG/CD8A/CD8B/IL7R/LCK/RFXAP | -1 |
| **hsa04640** | **Hematopoietic cell lineage** | 0.000 | 0.001 | CD2/CD3D/CD3G/CD5/CD8A/CD8B/CR2/IL7R | -1 |
| **hsa04660** | **T cell receptor signaling pathway** | 0.000 | 0.001 | CD3D/CD3G/CD40LG/CD8A/CD8B/LCK/PLCG1/RASGRP1 | -1 |
| **hsa04064** | **NF-kappa B signaling pathway** | 0.001 | 0.028 | BLNK/CD40LG/LCK/LTA/PLCG1/TRAF1 | -1 |

KEGG pathway enrichment results (P.adjust: BH method). Group: 1: Upregulated in the high-risk group; -1: Downregulated in the high-risk group.

## Supplementary Figure Legends

**Supplementary Figure 1. Detailed information about the influences of prior treatment and driver mutation on clinical responses.**

(A) Comparison of overall survival among the subgroups stratified by treatment lines in patients with low (0-49%) and high (50-100%) PD-L1 expression levels. Each column in the upper graphs shows the overall survival time of individual patients in the 6 subgroups classified by treatment line and PD-L1 expression level. The lower table shows the Cox hazard ratios between the subgroups stratified by treatment lines in the low (0-49%) and high (50-100%) PD-L1 expression groups. (B) The detailed information on clinical characteristics (age, sex, histology, and type of mutations), clinical responses (anti-tumor repose and OS) and treatments (molecular targeted or chemotherapeutic agents used in each treatment line) in NSCLC patients positive for driver gene mutations.

**Supplementary Figure 2. Correlation of PFAAs and metabolites before ICI therapy and cancer prognosis (overall survival)**

Kaplan-Meier analysis for overall survival in the groups stratified by the median concentration of 6 PFAAs and 3 metabolites, respectively. P value (log-rank test) and Cox hazard ratio (95% CI) are shown.

**Supplementary Figure 3. Prognosis and response to ICI therapy in 36 patients for transcriptome analysis of PBMCs**

(A) Kaplan-Meier estimates of overall survival in the high- (n = 6) and low-risk (n = 30) groups based on the multivariate model with PFAAs/metabolites in 36 patients included

in transcriptome analysis of PBMCs . P value (log-rank test) is shown. (B**)** The risk scores by the multivariate model with PFAAs/metabolites were compared using a Student's t-test between the subgroups classified by the anti-tumor responses (RECIST criteria).

**Supplementary Figure 4. Function enrichment of genes differentially expressed between the high- and low-risk groups**

(A) Dot plot of the top 10 GO terms from GO enrichment (P.< 0.05). Size of dots represent recruited counts of genes; increased dot diameters indicate increased gene numbers. Red color, higher significance; blue color, lower significance. Details are shown in Supplementary Table 3. (B) Bar plot of top 8 pathways from KEGG pathway enrichment (P < 0.05). Red: upregulated pathways in the high-risk group; Blue: downregulated pathways in the high-risk group. Bar length shows significance ($-\log_{10}P$). Details are shown in Supplementary Table 4. (C) GSEA analysis of Reactome pathways enrichment (P < 0.05). NES = normalized enrichment score. Nominal p-value calculated by the permutation test in GSEA analysis.

**Supplementary Figure 5. Function enrichment of amino acid metabolism-related genes differentially expressed between the high- and low-risk groups**

Plot of differentially expressed AAMGs performed by KEGG pathway enrichment analysis. Red: upregulated pathways in the high-risk group; Blue: downregulated pathways in the high-risk group. Pathways showing difference in expression with significance (P < 0.05, method: BH). Bar length showing significance ($-\log_{10}$ P).

**Supplementary Figure 6. Identification of amino acid metabolism-related genes**

**associated with patient prognosis.**

Kaplan-Meier estimates of overall survival in the subgroups stratified by RNA expression levels of AAMGs in PBMCs. Cutoff values between the high and low groups were set at the median of gene expression levels. P values (log-rank test) are shown.

**Supplementary Figure 7. Potential mechanisms of amino-acid metabolism in anti-tumor immunity in cancer patients treated with ICI**

The immune cells in the peripheral blood that circulated from the tumor microenvironment (TME) may reflect the composition, activity, and metabolic characteristics of immune cells within the TME. The multivariate model with PFAA/metabolite concentrations in the blood might be useful for inferring the amino acid metabolic characteristics of immune cells under the TME and systemic immune functions. Compared with the high-risk group, PBMCs in the low-risk group have characteristics similar to a "hot tumor" with a relatively high proportion of $CD8^+$ T cells and M1 macrophages and show differential expression of genes related to amino acid metabolism, including *SLC11A1*, *PHGDH*, and *HAAO*. Once patients in the low-risk group are treated with PD-1 inhibitors, they block PD-1 receptors on T cells, which circulate and infiltrate into the TME. As a result, T cells are rejuvenated from a state of immune exhaustion, resulting in activation of a series of T cell-mediated immune response, which include enhancing the interaction between macrophages and T cells, releasing liquid factors, and subsequently promoting killing of cancer cells. Meanwhile, patients in the low-risk group had a higher concentration of PFAAs such as tryptophan and serine, which provide sufficient energy and material requirements for immune cells.
